# Ionic Liquid Containing Block Copolymer Dielectrics: Designing for High-Frequency Capacitance, Low-Voltage Operation, and Fast Switching Speeds

**DOI:** 10.1021/jacsau.1c00133

**Published:** 2021-06-09

**Authors:** Alexander J. Peltekoff, Samantha Brixi, Jukka Niskanen, Benoît H. Lessard

**Affiliations:** † Department of Chemical & Biological Engineering, University of Ottawa, 161 Louis Pasteur, Ottawa, Ontario, Canada K1N 6N5; ‡ School of Electrical Engineering and Computer Science, University of Ottawa, 800 King Edward, Ottawa, Ontario, Canada K1N 6N5

**Keywords:** nitroxide-mediated polymerization (NMP), poly(ionic liquid), organic thin-film transistor (OTFT), electrolyte-gated transistor (EGT), block copolymer self-assembly, high capacitance dielectrics

## Abstract

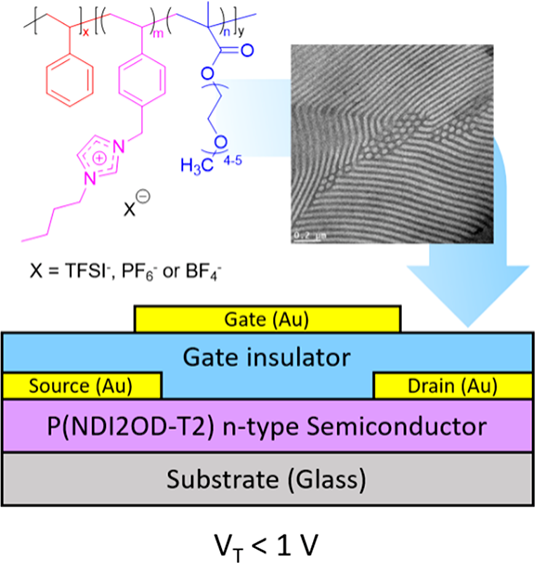

Polymerized ionic liquids (PILs) are a potential solution to the large-scale production of low-power consuming organic thin-film transistors (OTFTs). When used as the device gating medium in OTFTs, PILs experience a double-layer capacitance that enables thickness independent, low-voltage operation. PIL microstructure, polymer composition, and choice of anion have all been reported to have an effect on device performance, but a better structure property relationship is still required. A library of 27 well-defined, poly(styrene)*-b-*poly(1-(4-vinylbenzyl)-3-butylimidazolium-*random*-poly(ethylene glycol) methyl ether methacrylate) (poly(S)-*b*-poly(VBBI^+^[X]-*r*-PEGMA)) block copolymers, with varying PEGMA/VBBI^+^ ratios and three different mobile anions (where X = TFSI^–^, PF_6_^–^ or BF_4_^–^), were synthesized, characterized and integrated into OTFTs. The fraction of VBBI^+^ in the poly(VBBI^+^[X]-*r*-PEGMA) block ranged from to 100 mol % and led to glass transition temperatures (*T*_g_) between −7 and 55 °C for that block. When VBBI^+^ composition was equal or above 50 mol %, the block copolymer self-assembled into well-ordered domains with sizes between 22 and 52 nm, depending on the composition and choice of anion. The block copolymers double-layer capacitance (*C*_DL_) and ionic conductivity (σ) were found to correlate to the polymer self-assembly and the *T*_g_ of the poly(VBBI^+^[X]-*r*-PEGMA) block. Finally, the block copolymers were integrated into OTFTs as the gating medium that led to n-type devices with threshold voltages of 0.5–1.5 V while maintaining good electron mobilities. We also found that the greater the σ of the PIL, the greater the OTFT operating frequency could reach. However, we also found that *C*_DL_ is not strictly proportional to OTFT output currents.

## Introduction

Organic thin-film transistors (OTFTs) are carbon-based field-effect logic operators, which enable lightweight and flexible electronics through low-cost printing methods. An obstacle to the advancement of OTFT-based technologies is the need for low operating voltages. Currently, large-area printed batteries provide voltages, typically around 1.35 V, that are far too low to power typical OTFTs.^1^ Electrolytes are attractive gating materials as they provide an extremely high capacitance, responsible for the reduction of operating voltage that is independent of layer thickness, making them compatible with large scale printing technologies. Ionic liquids are room-temperature molten salts, which exhibit capacitances that are 2 orders of magnitude greater than conventional dielectrics due to the formation of electrical double layers (EDLs). The high capacitances lead to a greater number of charge carriers in the semiconductor channels, which results in high on-current and low threshold voltages.^2^ Unfortunately ionic liquids are problematic due to electrochemical doping of the semiconductor during operation leading to slow switching speeds.^3,4^ In addition, ionic liquids are liquids, which limits their potential for use in solid-state devices. Blending the ionic liquid (ion gels)^5^ with a polymer does lead to improved mechanical properties, but this does not reduce the potential for electrochemical doping. The ideal material should have the mechanical properties of a polymer combined with the performance of ionic liquids.

Polymerized ionic liquids (PILs) are solid electrolyte materials that exhibit the same EDL capacitances as ionic liquids but are unipolar, where only one ion is mobile, and the complementary charge is bound to the polymer backbone. When paired with an appropriate semiconductor, the possibility of electrochemical doping is eliminated as the EDL at the dielectric/semiconductor interface is formed by the large macromolecule that is too large to penetrate the semiconductor.^6^ Therefore, pairing polycation PIL (mobile anion) with an n-type semiconductor and polyanion PIL (mobile cation) with p-type semiconductors, prevents the possibility of faradaic oxidation and reduction of the organic semiconductor.^7^ The thickness-independent high-capacitances and elimination of electrochemical doping (faradaic effects) of unipolar PILs give them great potential to realize large scale production of low-power consuming OTFTs. However, PILs are plagued by sluggish response times due to their lower ionic conductivity compared to unpolymerized ionic liquids. There have been efforts to increase the solid-state ionic conductivity of PILs to improve OTFT response times when utilized as the gating mediums.^8−10^ For example, reducing the glass transition temperature (*T*_g_) of PIL leads to an increase in ionic conductivity.^11^ For example, poly(1-glycidyl-3-butylimidazolium bis(trifluoromethanesulfonyl)imide) supporting an ethylene glycol backbone exhibited ionic conductivity of 10^–5^ S cm^–1^ at 30 °C with a low-*T*_g_ of −14 °C.^12^ Intuitively, greater polymer chain flexibility facilitates ion transport through the materials in an analogous fashion to the correlation of viscosity and ionic conductivity for ionic liquids. Evidently, the ion dynamics and structural relaxation are strongly correlated above the *T*_g__,_ leading to a significant trade-off between ionic conductivity and mechanical integrity.^13,14^ Our group and others have shown that block copolymers exhibited ionic conductivities up to 2 orders of magnitude greater compared to random copolymers of similar compositions because of the microphase separation of the ionic segments forming conductive pathways through the material.^9,10,15^ While these studies examine the ionic conductivity of the polymer, this does not correlate directly to OTFT performance. For example an increased capacitance increases output currents, but decreases charge mobility (μ). This decreased μ is related to the way the EDL is formed by the charge carriers and electrolyte ions. Therefore, the choice of polymer, the mobile ion and the dielectric/semiconductor interface itself are critical, and no studies have systematically and simultaneously examined all these parameters.

In this study, we develop a series of 27 different block copolymers to probe the dielectric/semiconductor interface and the design of these critical materials. For example, we characterize the same polymer with different anions (TFSI^–^, PF_6_,^–^ BF_4_^–^) and their effect on device performance. In this example, the interface for each of these three devices is identical because the polymer is the same, and it is only the anion that migrates. However, our materials have a different capacitance which will influence μ. We have designed a library of poly(styrene)*-b-*poly(1-(4-vinylbenzyl)-3-butylimidazolium-*random*-poly(ethylene glycol) methyl ether methacrylate), (poly(S)-*b*-poly(PIL-*r*-PEGMA)), block copolymers with three different anions (TFSI^–^, PF_6_^–^, or BF_4_^–^). This will help elucidate structure–property relationships for the design of high performance PIL gating materials in n-type OTFTs and decouple the effect of ion conductivity, capacitance, and the device μ.

## Results and Discussion

**Scheme 1:**
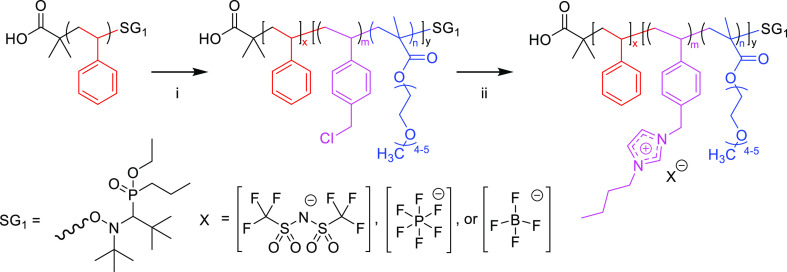
Synthesis of Poly(styrene)*-b-*poly(1-(4-vinylbenzyl)-3-butylimidazolium-*random*-poly(ethylene glycol) Methyl Ether Methacrylate), (Poly(S)-*b*-poly(VBBI^+^[X]-*r*-PEGMA)) Poly(Ionic Liquid) Gating Material Where X = TFSI^–^, PF_6_^–^ or BF_4_^–^. Reaction conditions: (i) Chain extension of a poly(S) macroinitiator using chloromethyl styrene (CMS) and poly(ethylene glycol) methyl ether methacrylate (PEGMA), *N*-tert-butyl-*N*-[1-diethylphosphono-(2,2-dimethylpropyl)] nitroxide (SG1), 1,4-dioxane, 90 °C, 24 h; (iia) butylimidazole, dimethylacetamide, 50 °C, 24 h; (iib) organic salt (LiTFSI, LiPF_6_, or NaBF_4_), dimethylacetamide, r.t., 48 h.

We synthesized a series of diblock copolymers consisting of a high glass transition temperature (*T*_g_) poly(styrene) block and a low *T*_g_ poly(ionic liquid) (PIL) containing second block (Scheme 1). The resulting materials were characterized, integrated into capacitor devices and studied by impedance spectroscopy to determine conductivity and double-layer capacitance. Finally, OTFT devices were fabricated, and electrical characterization was performed. The following explores the effect of molecular architecture and self-assembly on the resulting electrical performance.

### Block Copolymer Synthesis

All experimental conditions and formulations can be found in the Experimental Section (Table S1). Nitroxide mediated polymerization (NMP) is a controlled polymerization technique that can lead to well-defined molecular architectures without the need for transition metal catalysts or laborious purification techniques; making it an ideal technique for the development of functional polymers for printable electronics.^16−19^ Therefore, a single large batch of narrow dispersity and pseudoliving poly(styrene) (poly(S)) macroinitiator (*M̅*_w_/*M̅*_n_ = 1.06; *M̅*_n_ = 22.5 kg·mol^–1^, Table 1) was synthesized by NMP, ensuring to stop the reaction at low-conversion to maintain a high-degree of livingness, with a target number average molecular weight (similar to our previous works.^15,20,21^ Chain extension of the poly(S) macroinitiators was performed using chloromethyl styrene (CMS) and poly(ethylene glycol)methyl ether methacrylate (PEGMA) comonomers in varying comonomer feed ratios and with different target molecular weights (Table 1). We obtained nine different poly(S)-*b*-poly(CMS-*r*-PEGMA) diblock copolymers with unimodal molecular weight distributions ranging from *M̅*_w_/*M̅*_n_ = 1.07–1.24 with *M̅*_n_ values ranging from 24.5–39.6 kg·mol^–1^ (Table 1). Representative size exclusion chromatography (SEC) chromatograms of the poly(S) macroinitiator and the corresponding poly(S)-*b*-poly(CMS-*r*-PEGMA) diblock copolymers P100/0-8 and P25/75-4 are shown in Figure S1. The chromatograms illustrate a clear monomodal shift from the poly(S) macroinitiator to the final poly(S)-*b*-poly(CMS-*r*-PEGMA) demonstrating a higher *M̅*_n_. Further examination of the trace from chain-extended products of high methacrylate content (shown in red) exhibit a slight tailing on the leading edge, which is indicative of termination by combination resulting in a broadening of molecular weight distributions.^22^ This slight broadening is consistent with typical chain extensions by NMP^23,24^ using PEGMA and other methacrylates. The final molar composition of PEGMA, CMS, and styrene (*F*_PEGMA_, *F*_CMS_, and *F*_S_) of the block copolymers were determined by ^1^H NMR and are presented in Table 1; a representative spectrum detailing composition calculation is presented in the Supporting Information (Figure S2). The composition of these second blocks varied between 100:0 (pure CMS) to 23:77 (mostly PEGMA) (Table 1). A small trend can be seen where the CMS integrated into the polymer is slightly higher than the feed, suggesting CMS preferentially reacts with itself and PEGMA because of the favorable reactivity ratios. This was not a surprise as we have previously shown CMS and other styrenic monomers are controlling monomers in the NMP of methacrylates.^25,26^ Overall, the pseudoliving poly(S) macroinitiator was effective in producing a library of poly(S)-*b*-poly(CMS-*r*-PEGMA) precursor block copolymers with relatively narrow, monomodal molecular weight distributions.

**Table 1:** Molecular Weight Distribution Data and Glass Transition for Poly(S)-*b*-poly(CMS-*r*-PEGMA) Diblock Copolymer Precursors to Ionic Liquid Containing Block Copolymers

Exp. ID^a^	*∂n*/*∂c*^b^ (mL g^–1^)	*M̅*_n_^2^ (kg mol^–1^)	*Đ*^2^ (*M̅*_w_/*M̅*_n_)	*F*_STY_^c^	*F*_CMS_^c^	*F*_PEGMA_^c^	*R*_CMS/PEGMA_^c^	*T*_g_^d^ [X] (°C)
poly(S)	0.192	22.5	1.06	1.00				100
P25/75-2	0.190	27.0	1.10	0.86	0.10	0.04	0.88	TFSI^–^ (99); PF_6_^–^(99); BF_4_^–^(99)
P25/75-4	0.096	58.2	1.10	0.88	0.04	0.09	0.41	TFSI^–^ (99); PF_6_^–^(99); BF^–^99)
P25/75-6	0.179	28.0	1.07	0.95	0.02	0.03	0.64	TFSI^–^ (99); PF^–^(99); BF_4_^–^(99)
P50/50-4	0.194	31.8	1.24	0.69	0.17	0.14	1.17	TFSI^–^ (−7,106); PF_6_^–^(21,105); BF_4_^–^(17,105)
P50/50-6	0.167	35.9	1.23	0.67	0.18	0.15	1.27	TFSI^–^ (−7,106); PF_6_^–^(22,105); BF_4_^–^(19,106)
P75/25-6	0.246	27.1	1.17	0.69	0.23	0.08	2.85	TFSI^–^ (2,107); PF_6_^–^(55,106); BF_4_^–^(48,105)
P75/25-8	0.202	31.4	1.12	0.74	0.20	0.06	3.17	TFSI^–^ (2,105); PF_6_^–^(52,105); BF_4_^–^(49,103)
P100/0-4	0.235	24.5	1.12	0.81	0.18	-	-	TFSI^–^ (12,105); PF_6_^–^(101); BF_4_^–^(98)
P100/0-8	0.154	39.6	1.17	0.70	0.30	-	-	TFSI^–^ (11,106); PF_6_^–^(104); BF_4_^–^(99)

a Experimental identification (Exp. ID) we define the as P*X*/*Y*-*Z* where *X*/*Y* = the target ratio of chloromethylstyrene (CMS) versus poly(ethylene glycol) methyl ether methacrylate (PEGMA) and where *Z* corresponds to the weight of total comonomers (CMS + PEGMA) added to 1 g of macroinitiator in chain extension polymerizations.

b The *∂n*/*∂c* was determined by offline batch dRI measurements for each polymer. Number-average molecular weight (*M̅*_n_), weight-average molecular weight (*M̅*_w_), and the dispersity (*M*_w_/*M*_n_) were determined by size exclusion chromatography (SEC).

c Molar compositions were determined by ^1^H NMR. *R*_CMS/PEGMA_ is the molar ratio of CMS relative to PEGMA in the second block.

d Glass transition temperature (*T*_g_) determined by differential scanning calorimetry (DSC) on the same base polymer with varying salts [X] = TFSI^–^, PF_6_^–^, or BF_4_^–^.

The final PIL-containing block copolymers were prepared by subsequent quaternization and anion exchange reactions on the precursor polymers (Scheme 1). First, the quaternization reaction on the CMS moiety with 1-butylimidazole was performed leading to poly(styrene)-*b*-poly(1-(4-vinylbenzyl)-3-butylimidazolium-*r*-poly(ethylene glycol) methyl ether methacrylate), (poly(S)-*b*-poly(VBBI^+^-*r*-PEGMA)) block copolymers; this reaction mixture was then divided into three, so a subsequent anion exchange with 3 different organic salts was performed poly(S)-*b*-poly(VBBI^+^[X]-*r*-PEGMA) (where [X] = BF_4_^–^, PF_6_^–^, or TFSI^–^). These anions were selected for their delocalized charge and size which increases conductivities.^27^ Isolation of the intermediate product was intentionally avoided as it could not be effectively obtained by precipitation and dialysis methods resulting in low yields. However, after substituting the Cl^–^ anion with the organic anions (TFSI^–^, BF_4_^–^, PF_6_^–^), the macromolecule becomes completely insoluble in water, facilitating the isolation of the final product, which was purified by repetitive washing with water. A total of 27 final poly(S)-*b*-poly(VBBI^+^[X]-*r*-PEGMA) block copolymers (Table 1) possessing three different mobile anions; X = BF_4_^–^, PF_6_^–^, and TFSI^–^ were obtained and confirmed by ^1^H NMR (see Experimental Section and Supporting Information).

### Self Assembly of Poly(Ionic Liquid) Block Copolymers

**Figure 1:**
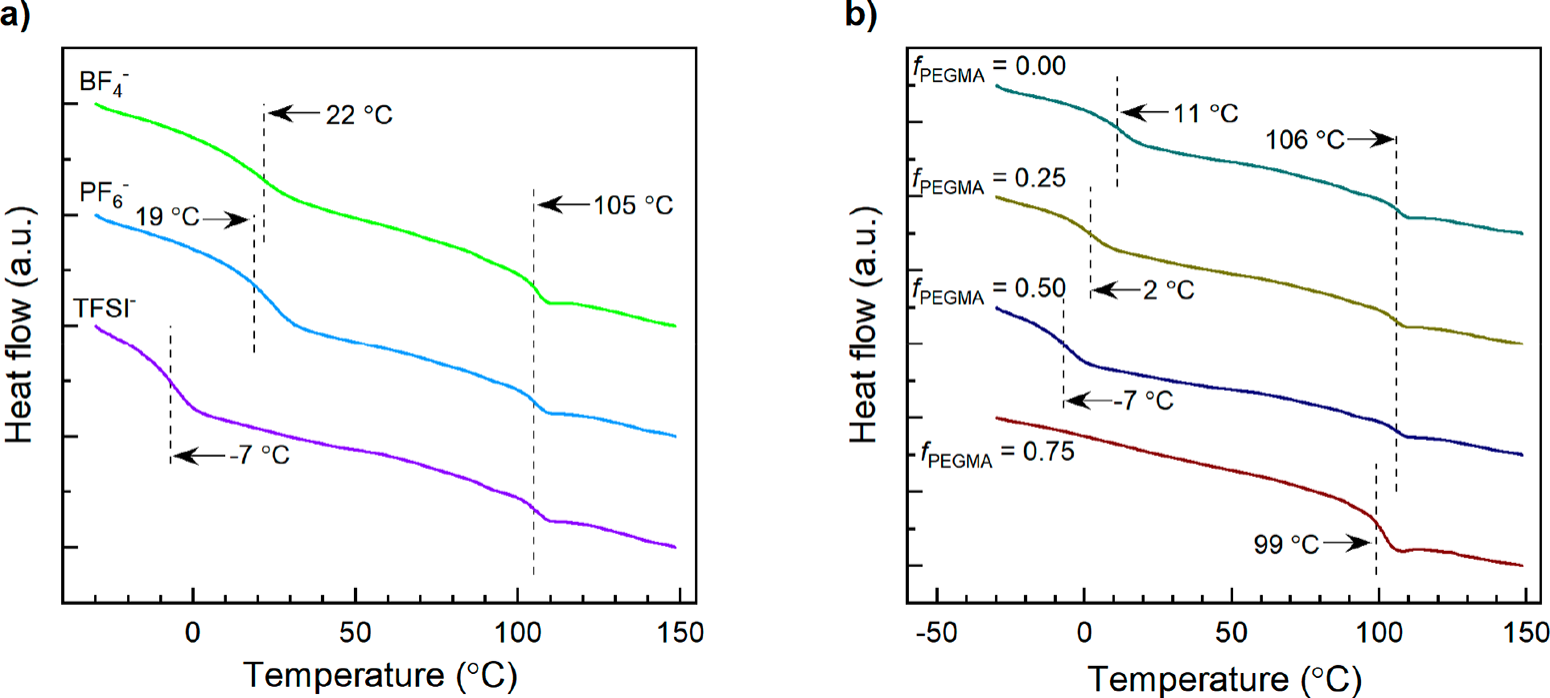
Characteristic differential scanning calorimetry (DSC) thermograms of poly(S)-*b*-poly(VBBI^+^[X]-*r*-PEGMA) (where [X] = BF_4_^–^, PF_6_^–^, or TFSI^–^) block copolymers where (a) compares the effect of 3 different salt forms, TFSI^–^, PF_6_^–^, and BF_4_^–^, within the same polymer P50/50 and (b) compares the same salt, TFSI^–^, on 4 different block copolymers with different PEGMA loadings, P100/0-8, P75/25-8, P50/50-6, and P25/75-6. The thermograms are from the second heat cycle in a heat/cool/heat experiment. The glass transition temperatures (*T*_g_) for all materials are summarized in Table 1.

All PIL-containing block copolymers were first characterized by differential scanning calorimetry (DSC) and characteristic thermograms can be found in Figure 1. The glass transition temperature (*T*_g_) of the materials was influenced by both the composition of the poly(VBBI^+^[X]-*r*-PEGMA) block and the nature of the mobile anion. Block copolymers with an *R*_CMS/PEGMA_ > 0.88 (Table 1) exhibited two distinct *T*_g_ values: one corresponding to the poly(S) block and a second corresponding to the poly(VBBI^+^[X]-*r*-PEGMA) block. Two distinct transitions suggests phase separation is occurring.^28^ Block copolymers containing >75 mol % PEGMA displayed only one *T*_g_ that was below that of the poly(S) macroinitiator, suggesting the blocks are miscible and no phase separation is taking place (Figure 1).^28^ The selection of anionic salt affected the *T*_g_ of the poly(VBBI^+^[X]-*r*-PEGMA) block, where TFSI^–^ containing polymers exhibited the lowest *T*_g_, followed by PF_6_^–^ and BF_4_^–^ (Figure 1, Table 1). This observation is consistent with studies that have shown the melting temperature of an ionic liquid decreases with increasing ion diameter.^29^ It is interesting to note that both poly(S)-*b*-poly(VBBI^+^[X]) block copolymers (i.e., *F*_PEGMA_ = 0) with PF_6_^–^ and BF_4_^–^ anions exhibited only one *T*_g_, while the same polymer with TFSI^–^ led to two *T*_g_ values (Table 1). This suggests that not only polymer composition but also the choice of the anion play a critical role in the self-assembly of the resulting block copolymers.

**Figure 2:**
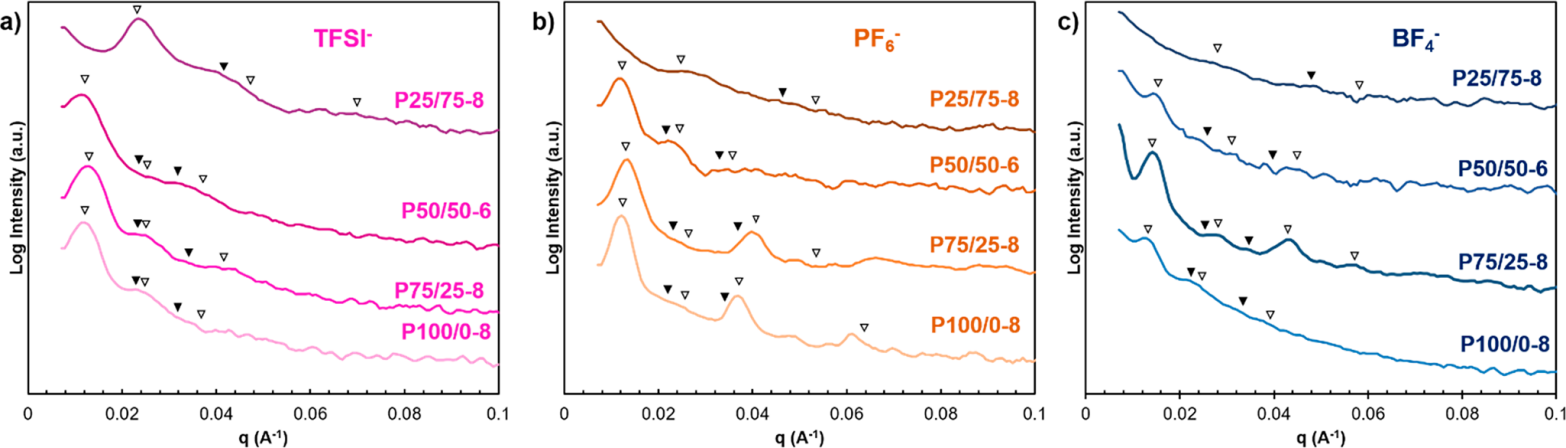
Through-plane small-angle X-ray scattering profiles of poly(S)-*b*-poly(PIL-*r*-PEGMA) block copolymers functionalized with (a) TFSI^–^, (b) PF_6_^–^, and (c) BF_4_^–^. The inverted filled triangles (▼) of samples indicate expected peak positions at q*, √3q*, 2q*, and √7q* for hexagonally packed cylindrical (HEX) morphology. The inverted open triangles (▽) indicate expected peak positions at q*, 2q*, 3q*, and 4q* for lamellar (LAM) morphology.

**Figure 3:**
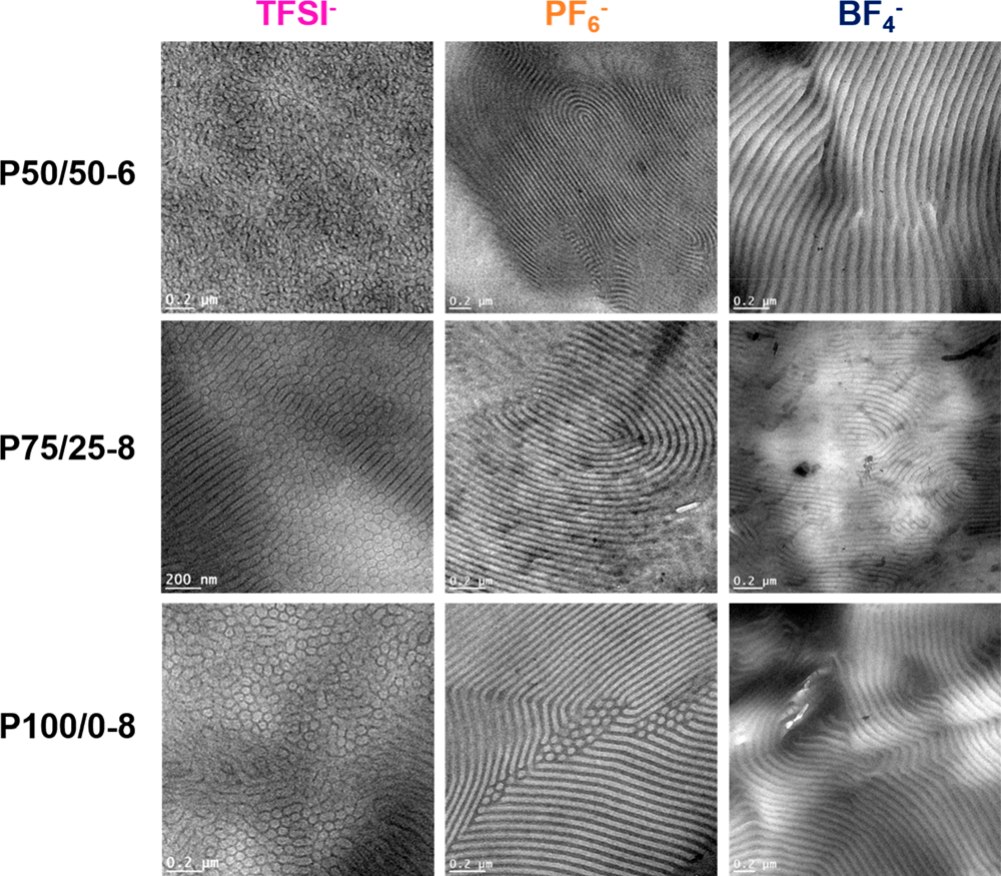
Transmission electron microscopy (TEM) images of poly(S)-*b*-poly(VBBI^+^[X]-*r*-PEGMA) copolymers where the rows represent the polymer P50/50-6 (top), P75/25-8 (center), and P100/0-8 (bottom) and where the columns represent the choice of anions: TFSI^–^ (left), PF_6_^–^ (center), and BF_4_^–^ (right). All samples were solvent cast from methyl ethyl ketone (MEK) over ∼24 h at 130 °C in vacuum. Dark microdomains in TEM correspond to the poly(VBBI^+^[X]-*r*-PEGMA) microphase.

To study the effect of morphology on OTFT performance, the molecular architecture and choice of anion were characterized as a function of degree of microphase separation. The morphology of 12 characteristic PIL block copolymers that demonstrated dual *T*_g_ values (Table 1) were evaluated by both through-plane small-angle X-ray scattering (SAXS) and transmission electron microscopy (TEM), Figures 2 and 3, respectively. Prior to morphological characterization materials were annealed to allow the self-assembled block copolymers to attain a thermodynamic equilibrium in regard to their organization.^30^ In all cases, microphase ordering with varying long-range periodicity are observed by SAXS. Variations in peak intensity as well as location suggests changes in morphology from lamella (LAM) to hexagonally packed cylinders (HEX) and mixtures of both. For example, samples exhibit the characteristic reflections at q*, 2q*, 3q*, 4q*, and 5q* corresponds to LAM, while reflections at q*, q*√3, 2q*, q*√7, and 3q* corresponds to HEX. Similar patterns have been reported for other PIL-containing block copolymers.^8,31^

The corresponding structures are represented in Table 2. In general, the q* decreases, corresponding to an increased domain sizes (*d* = 2π/q*), as the anion size increase BF_4_^–^ < PF_6_^–^< TFSI^–^ (Table 2). P25/75-8 with BF_4_^–^ and PF_6_^–^ exhibited a broad scattering peak indicating weaker microphase separation without long-range periodicity. This weak long-range microphase separation (WLO) is likely due to the partial miscibility of the two blocks,^9^ which can be seen in the DSC traces where only one *T*_g_ is present (Table 1). P25/75-8 with TFSI^–^ exhibited a strong q* scattering peaks with broad 2q* and √3q* suggesting more significant microphase separation into a mixture of LAM and HEX compared to the same polymer with PF_6_^–^ or BF_4_^–^. In all three cases, the calculated domain size was on the order of 20–26 nm and the TEM images show no significant long-range order. All other polymers (P50/50-6, P75/25-8, and P100/0-8 with either anions) exhibited relatively larger domain sizes ranging from 42 to 52 nm and clearly visible long-range order by TEM (Figure 3). Ye at al. observed an increase in domain size as PIL block increased relative to poly(MMA) block (i.e., poly(MMA) block remained the same length^9^ and the PIL block size increased), while our study shows no correlation between PIL composition and domain size, likely due to the negligible changes in relative block length (i.e., as PIL content increase in our study, PEGMA content decreases keeping total block length comparable). P100/0-8 with BF_4_^–^ and all block copolymers that were functionalized with TFSI^–^ (Figure 2) experienced higher-order reflections at q*√3 and q*√7 indicative of HEX morphology, which is consistent with the TEM images. Both P75/25 and P100/0-8 with PF_6_^–^ and P75/25-8 with BF_4_^–^ exhibited strong q* scattering peaks at q*, 3q* and 5q* with structure factor extinctions of 2q* and 4q*, indicative of a LAM morphology with apparent symmetric volume fractions of each block.^8^ The LAM morphology was supported using TEM. P50/50-6 with PF_6_^–^ exhibited strong features at 2q*, which was confirmed to correspond to LAM by TEM (Figure 3). P100/0-8 with PF_6_^–^ exhibited strong features suggesting LAM morphology. However, small features at q*√3 and q*√7 indicative of HEX morphology were also detected by SAXS and the combination of HEX and LAM was also detected using TEM. These results indicate that the library of PIL block copolymers, with two distinct *T*_g_ values exhibit self-assembled microstructures with long-range order and that the domain size and organizational structure (HEX vs LAM) depends on block copolymer architecture and choice of anion. This self-assembly clearly demonstrates well-defined domains comprised of the low *T*_g_ PIL blocks, which can lead to improved anion transport through the film. This film morphology will therefore directly affect the device performance.

**Table 2:** Domain Size and Long Range Order Morphology as Determined by SAXS

exp. ID^a^	anion	domain size (nm)^b^	morphology^c^	σ (S cm^–1^)^d^
P25/75-8	BF_4_^–^	21.7	WLO	
P50/50-6	BF_4_^–^	42.1	LAM	7.9 × 10^–8^
P75/25-8	BF_4_^–^	44.2	LAM	1.2 × 10^–8^
P100/0-8	BF_4_^–^	48.7	LAM+HEX	4.6 × 10^–9^
P25/75-8	PF_6_^–^	22.7	WLO	
P50/50-6	PF_6_^–^	52.4	LAM	4.1 × 10^–9^
P75/25-8	PF_6_^–^	46.5	LAM	5.8 × 10^–11^
P100/0-8	PF_6_^–^	49.1	LAM + HEX	7.4 × 10^–11^
P25/75-8	TFSI^–^	26.8	HEX	3.6 × 10^–8^
P50/50-6	TFSI^–^	52.0	HEX	3.4 × 10^–7^
P75/25-8	TFSI^–^	47.8	HEX	1.7 × 10^–7^
P100/0-8	TFSI^–^	52.0	HEX	1.4 × 10^–9^

a Experimental identification (exp. ID) we define the as P*X*/*Y*-*Z* where *X*/*Y* = the target ratio of chloromethyl styrene (CMS) versus poly(ethylene glycol) methyl ether methacrylate (PEGMA) and where *Z* corresponds to the weight of total comonomers (CMS + PEGMA) added to 1 g of macroinitiator in chain extension polymerizations.

b Domain sizes (*d* = 2π/q*) determined by small-angle X-ray scattering.

c Morphology: Hexagonally packed cylindrical (HEX) morphology, lamellar (LAM) morphology, and weak long-range microphase separation (WLO).

d Ionic conductivity (σ) was estimated using σ = *d*/(*R*_b_*A*), where *d* is the film thickness, *R*_b_ is the bulk resistance, and *A* is the area of the capacitor. Conductivity and capacitance were determined by electrical impedance spectroscopy (EIS).

### Metal–Insulator–Metal Capacitors

**Figure 4:**
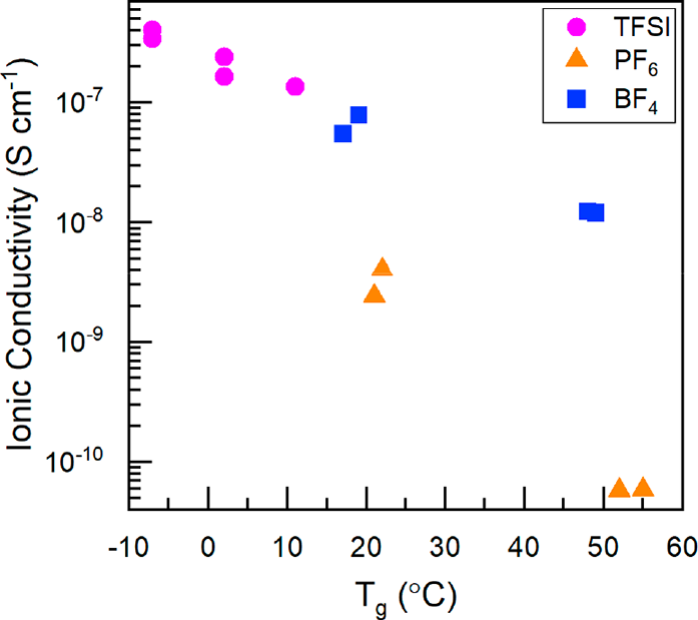
Conductivity versus the lower glass-transition temperature (*T*_g_) of the poly(S)-*b*-poly(VBBI^+^[X]-*r*-PEGMA) (where [X] = BF_4_^–^, PF_6_^–^, or TFSI^–^) block copolymers. All experimentally obtained *T*_g_ are tabulated in Table 1.

The poly(S)-*b*-poly(VBBI^+^[X]-*r*-PEGMA) (where [X] = BF_4_^–^, PF_6_^–^, or TFSI^–^) block copolymers were used as the insulating layer in the fabrication of metal–insulator–metal capacitors and characterized using electrochemical impedance spectroscopy (EIS). The ionic conductivity (σ) was estimated using σ = *d*/(*R*_b_*A*), where *d* is the film thickness, *R*_b_ is the bulk resistance, and *A* is the area of the capacitor. Nyquist plots (Figure S4) are obtained from the impedance data for each block copolymer. The *R*_b_ was determined by the radius of a circle fit to the ionic segment of the Nyquist plot produced from the EIS experiments. The resulting σ obtained for the block copolymers, which exhibited measurable conductivity, were plotted as a function of the lower *T*_g_, which corresponds to the *T*_g_ of the PIL-containing block (Figure 4).

**Figure 5:**
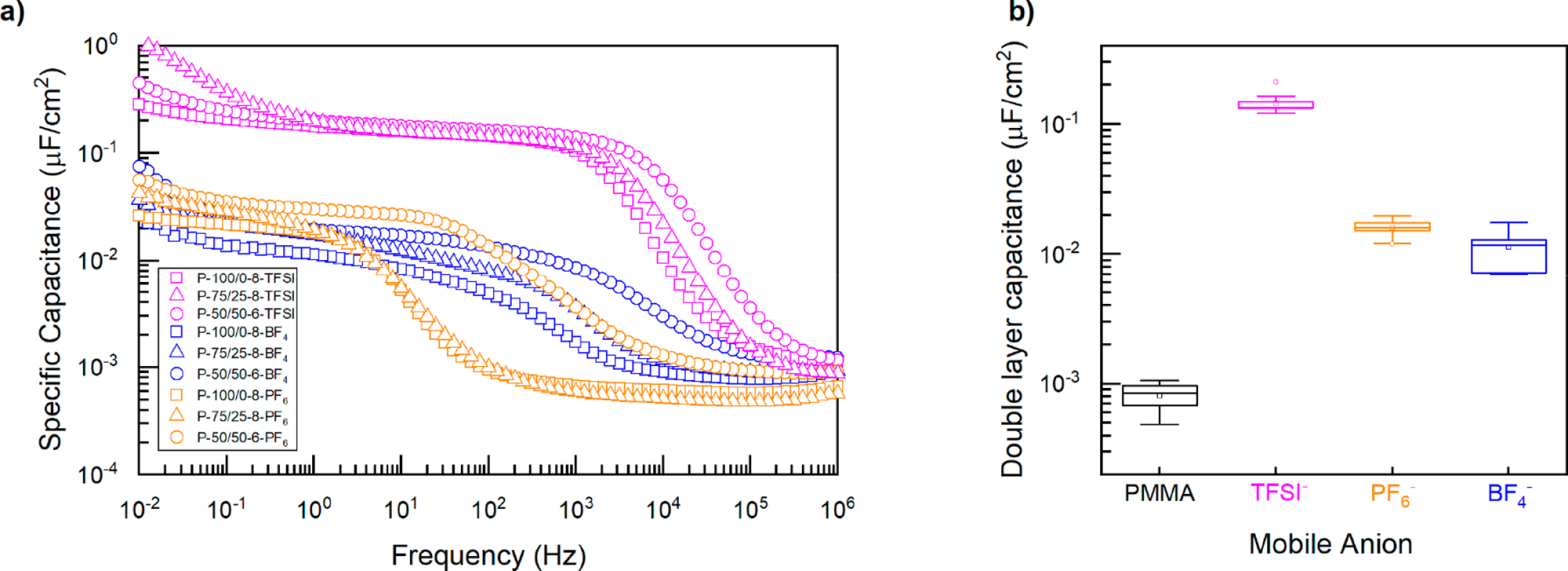
(a) Representative specific capacitance versus frequency curves and (b) double layer capacitance for poly(S)-*b*-poly(VBBI^+^[X]-*r*-PEGMA) (where [X] = BF_4_^–^, PF_6_^–^, or TFSI^–^) in MIM capacitors. The data in panel b is presented in a standard box plot convention, where the upper and lower lines of the box represent the 75th and 25th percentile, while the middle horizontal lines represent the median. The outer whiskers represent the upper and lower quartile +1.5 the interquartile range. Additionally, two symbols were used in the data representations (square and circle): the square represents the mean, whereas the circle represents outliers.

In all cases, the σ increases as the *T*_g_ of the PIL block decreases resulting in a 3 orders of magnitude increase in σ for a 65 °C drop in *T*_g_ (Figure 4), a well established relation with this class of materials.^27,32^ This observation extends from ionic liquids, where Angel et al.^33^ found the σ of neat ionic liquids followed an inverse linear dependence on the viscosity, which is seemingly independent of the cation and anion. Observed discrepancies from this ideal dependence have been attributed to differences in the ion sizes; however, most ionic liquid species in practice possess similar volumes.^34,35^ The polymer architecture of similar PEO-based single-ion conducting polymer electrolytes on conductivity has been explored. For example, Mapesa et al. showed the incorporation of a long alkyl spacer between charged imidazolium moieties and polymer backbones offered extra mobility to the ion pairs, leading to an increase in ionic conductivity.^36^ This placement of the imidazolium moiety was further explored by Kuray et al., who found that materials with the charged moiety placed in a pendant had a greater σ than those with the functionalization directly in the polymer backbone.^37^ Choi et al. explored the difference in σ between materials with an alkyl spacer versus PEO spacers and showed the PEO-containing PILs were significantly more conductive because of the relatively flexible and polar nature of the PEO spacer that behaves as a plasticizer and solvator.^38,39^ Other PEO structures have also been explored, such as linear side chain versus cyclic side-chain,^40^ as well as utilizing PEO as the polymeric backbone with imidazolium pendant moieties.^12^ Ikeda et al. explored the length of tail off of the imidazolium pendant and found it has no major influence on *T*_g_ or conductivity.^41^ We found that the effect of *T*_g_ was more significant than the ionic concentration itself, i.e. composition of the poly(VBBI^+^[X]-*r*-PEGMA) block. These results are consistent with the SAXS and TEM results, suggesting that low *T*_g_ pathways through the film improve anion transport and, therefore, increase ionic conductivity. This corroborates observations by Chen et al. with butyl methacrylate imidazolium based PIL homopolymers with TFSI^–^ and BF_4_^–^ anions who showed that reduced *T*_g_ has a more significant effect on σ than the overall charge content.^11^ Further examination into the influence of *F*_PEGMA_ for the poly(VBBI^+^[X]-*r*-PEGMA) block, we can see that σ increases from *F*_PEGMA_ = 0 (pure PIL, P100/0-4, P100/0-8) to *F*_PEGMA_ = 0.15 (P50/50-4, P50/50-6). Interestingly, when *F*_PEGMA_ > 0.75, this trend no longer holds true. The resulting materials exhibited no σ (with the exception of P25/75-4-TFSI), which is likely due to unfavorable morphology as demonstrated by only exhibiting a single *T*_g_ and negligible self-assembly by TEM. These results demonstrate that block copolymer architecture and microphase separation both play a critical role in obtaining high *σ.*Figure 4 further demonstrates that the choice of anion plays a significant role on the *T*_g_ and the resulting σ. In all cases a trend of σ, TFSI^–^ > BF_4_^–^> PF_6_^–^, was observed. A similar trend was reported by Chen et al. for similar anions when using methacrylate based imidazolium PIL homopolymers with alkyl spacers.^42^ Ganesan and co-workers have attributed ion size as a handle on σ within the respective physicochemical ion classes of PILs, while noting that large ions with delocalized charge can outperform some smaller ions, this corresponds to our observations where the TFSI materials exhibited the highest conductivity.^43^ In addition to σ, the electrical double-layer (EDL) capacitance is a critical characteristic necessary to understand prior to integration into organic thin film transistor (OTFT) devices. Very few reports exist on the characterization of ionic liquid EDL capacitance and even less on the characterization of PILs EDL capacitance. For example, typical electrolytes contain a solvent dipole and supporting electrolyte that are well understood using Helmholtz and Guoy–Chapman models.^44^ Unlike typical electrolytes, ionic liquids interfacial phenomena greatly effect of the EDL because of their direct contact with the electrode. Therefore, electrode properties, such as work function and surface roughness, have been shown to affect ionic liquid EDL capacitance, suggesting the importance of the interface.^45^ However, it is established that polarizability and ion size affects the EDL.^46^ For example, an increase in molecular volume will increase the static dielectric constant.^46^ Colby and co-workers showed that the capacitance of imidazolium ionic liquids is increased when polymerized into PILs.^46^ The specific capacitance as a function of frequency and the corresponding average EDL capacitance for the poly(S)-*b*-poly(VBBI^+^[X]-*r*-PEGMA) block copolymers as a function of anion ([X] = BF_4_^–^, PF_6_^–^, or TFSI^–^) are plotted in Figure 5. The specific capacitance shown at intermediate frequencies (∼10^–3^–10^–1^ Hz) is associated with the EDL capacitance. It is clear that the magnitude of EDL capacitance is dictated by the choice of mobile anion, where TFSI^–^ producing the greatest value followed by PF_6_^–^ and BF_4_^–^. These results are consistent with the trend observed by Fujimoto et al. with ionic liquids based on 1-butyl-3-methylimidazolium (BMIM) cation.^47^ At high frequencies (prior to EDL formation), all materials exhibited similar capacitances (Figure 5a). Choi et al. have shown that the capacitance is increased when the cation is physically further from the polymer backbone by use of a spacer, facilitating a response to an applied electric field, increasing their relaxation strength and, thus, dielectric constant; however, this increase is only seen in the high-frequency regime, prior to the EDL formation.^46^ At lower frequencies block copolymers with elevated *F*_PEGMA_, experienced a small secondary rise in capacitance (Figure 5a). This low frequency rise suggests an orientation of PEGMA chains or of the alkyl group on butyl-imidazole^48,49^ Another important result of the formation of the EDL layer is that it provides a thickness independent dielectric, which is critical for the integration of these materials into large-scale commercial printed electronics. To confirm that these block copolymers exhibited thickness-independent capacitance, we characterized ∼400 and ∼800 nm films of all block copolymers and obtained identical results (Figure S5).

The EDL capacitances achieved for these TFSI^–^-based block copolymers were on the order of 200–300 nF·cm^–2^ (Figure 5, Table 2), which is lower than that of other TFSI^–^-based PILs we have studied using the same characterization setup.^15,50^ Previously, we reported triazole-based styrenic homopolymers^50^ and poly(methyl methacrylate*-r-*styrene)*-b-*poly(VBBI^+^TFSI^–^) (poly(MMA-*r*-S)-*b*-poly(VBBI^+^TFSI^–^) block copolymers,^15^ which were both characterized by an EDL capacitance of 1000–2000 nF·cm^–2^. It is also important to note that, in both cases, those polymers were found to have a greater *T*_g_ and a reduced σ compared to these poly(S)-*b*-poly(VBBI^+^[X]-*r*-PEGMA) block copolymers. Therefore, high conductivity does not necessarily lead to high EDL capacitance. This comparison also illustrates that, while the choice of anion is important, the molecular architecture, the self-assembly, the interfacial composition, and the EDL formation are critical for high capacitance.

### Poly(Ionic Liquid)-Gated Thin-Film Transistors

**Figure 6:**
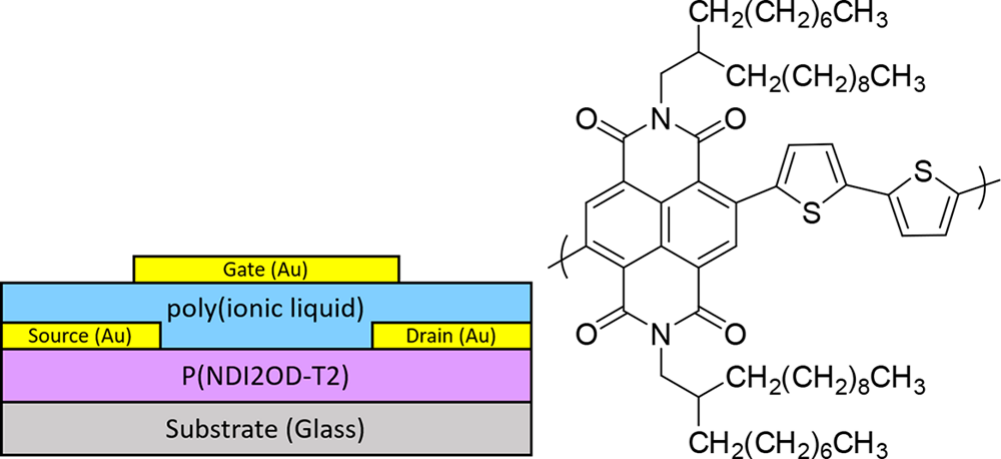
Schematic cross section of TGTC transistors based on poly(NDI2OD-T2) n-type semiconductor, poly(S)-*b*-poly(VBBI^+^[X]-*r*-PEGMA) (where [X] = BF_4_^–^, PF_6_^–^, or TFSI^–^) as poly(ionic liquid) gating medium, and gold contacts.

To ensure only electrostatic gating is present, which does not affect the band structure of the semiconductor as electrochemical doping does, ions in the electrolyte must only interact with the charge in the channel via Coulombic interactions.^51^ One method to ensure electrostatic gating while maintaining the benefits of high capacitances from electrolyte gating is the use of single-ion conductors which, when properly paired with an appropriate semiconductor, will result in only electrostatic gating.^52^ Furthermore, Fullerton-Shirey and co-workers have shown single-ion conductors (a mobile cation paired with a p-type semicondcuctor) can achieve charge carrier densities in OTFTs of equivalent magnitude to that of dual ion conductors.^53^ In this study, top-gate top-contact (TGTC) transistors were fabricated using P50/50-6 (Table 1), a poly(S)-*b*-poly(VBBI^+^[X]-*r*-PEGMA) materials functionalized with either [X] = TFSI^–^, PF_6_^–^, or BF_4_^–^ as the mobile anions; gold contacts, and solution processable poly{[N,N′-bis(2-octyldodecyl)-naphthalene-1,4,5,8-bis(dicarboximide)-2,6-diyl]-alt-5,5′-(2,2′-bithiophene)} (poly(NDI2OD-T2))^54^ n-type semiconductor (Figure 6, Table S4). It is important to note that we paired a PIL with mobile anion with the complementary n-type semiconductor to avoid electrochemical doping. Polymer layers were formed via spin-coating in atmospheric conditions and contacts were deposited by physical vapor deposition (PVD) under vacuum (see Experimental Section). It is important to note that the EDL capacitance is a function of the ionic conductivity and dielectric constants of the materials, as well as the electrode geometry and type,^55−57^ this is why in all cases the same electrode materials were used for the MIM capacitors, as well as the OTFTs. Furthermore all poly(ionic liquid) materials were compared under the same conditions using the same electrode geometry and processing parameters to ensure adequate comparison. The top-contact architecture was selected in an effort to reduce contact resistance exhibited in similar devices from our previous work which utilized ITO contacts in a bottom-contact architecture.^15^ High-contact resistance is commonly seen in OTFTs with bottom-contact configurations and from energetic mismatched semiconductor-electrode metal pairs.^58^ The unipolar PIL gating materials were purposefully paired with an n-type semiconductor to prevent electrical doping of the semiconductor with the mobile ions during device operation. When a positive voltage is applied to turn on the device, the negative anions move to form an EDL at the gate; this leaves the semiconductor/PIL interface to consist solely of the large polycation backbone, which is simply too big to penetrate the permeable semiconductor.^59^ Since all materials were obtained from the same precursor block copolymer material (P50/50-6); this semiconductor/PIL interface should be identical for *all* devices fabricated irrespective of their mobile anion making any observed differences in drain-currents solely due to the effect of the mobile anion. To the best of our knowledge, this would be the only report of the identical polymer with different anions being used as gating material for n-type OTFTs. Small changes in polymer structure (composition, molecular weight, polydispersity) can have significant effect on self-assembly and EDL formation making the comparison of different systems quite challenging; which again is avoided here by having the identical polymer precursor.

**Figure 7:**
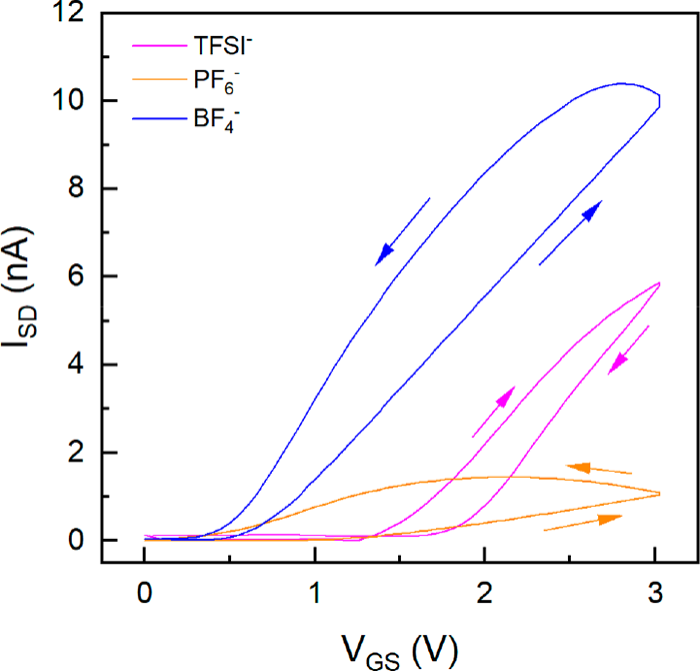
Representative transfer characteristics in the linear regime (*V*_DS_ = 1 V) of poly(S)-*b*-poly(VBBI^+^[X]-*r*-PEGMA) (where [X] = BF_4_^–^, PF_6_^–^, or TFSI^–^) OTFTs. The gate voltage was swept linearly at a rate of 250 mV/s (*L* = 30 μm, *W* = 1000 μm).

**Figure 8:**
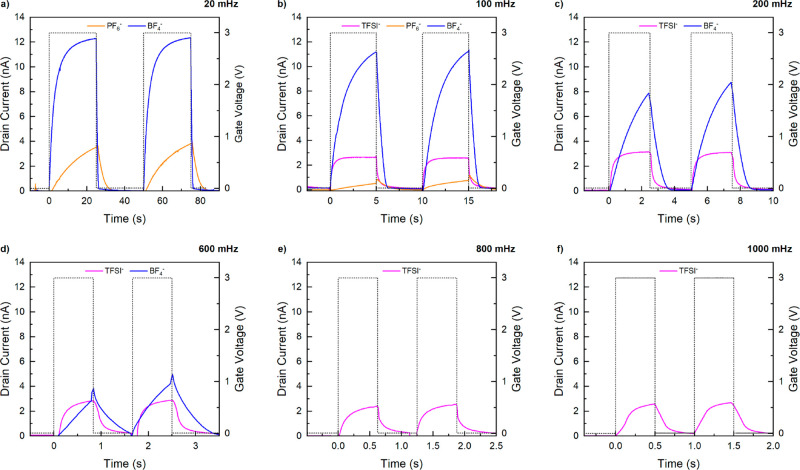
Frequency-dependent measurements of P50/50-6 a poly(S)-*b*-poly(VBBI^+^[X]-*r*-PEGMA) (where [X] = BF_4_^–^, PF_6_^–^, or TFSI^–^) gated OTFTs, where the traces correspond to the anion as TFSI^–^ (magenta), PF_6_^–^ (orange), and BF_4_^–^ (blue). The drain current response to a 3 V gate voltage applied in a two-square wave burst at frequencies ranging from 20 to 1000 mHz.

Figure S6 shows the drain current (*I*_DS_) versus drain voltage (*V*_DS_) output curves for poly(NDI2OD-T2) devices (*W*/*L* = 1 mm/30 μm) fabricated with P50/50-6 using TFSI^–^, PF_6_^–^, or BF_4_^–^. P50/50-6 was selected as a point of comparison because of its high ionic conductivity. The output characteristics of all the transistors exhibit linear source-drain behavior up to 1 V, where TFSI^–^ and BF_4_^–^ devices start to display saturation behavior. While the resulting devices displayed some degree of contact resistance; they represented a significant improvement over our earlier studies with similar dielectric materials in a top gate bottom contact architecture.^15^ The greatest *I*_DS_ were obtained from devices made using P50/50-6 with the BF_4_^–^ anion, followed by TFSI^–^ and PF_6_^–^, respectively (Figures 7 and 8). These results indicate that the choice of anion plays a role on the device current even when the devices all had identical polycation backbone and semiconductor interface. Note that the anion moves away from the interface during operation and because we used the identical cation polymer for each device the interface should be identical. Additionally, the relative trend of *I*_DS_ obtained from these devices does not correspond to EDL capacitance values observed in capacitors where TFSI^–^ > PF_6_^–^ > BF_4_^–^.

The transfer characteristics for the OTFTs were obtained by sweeping the gate voltage (*V*_GS_) at a sweep rate of approximately 250 mV/s to ensure EDL formation while holding the *V*_SD_ constant at 1 V. All materials displayed hysteresis, which is commonly observed in electrolyte dielectrics as a consequence of the relatively slow movement of ions and moisture.^60−62^ Several parameters were extracted by aid of linear fit to the transfer curves: the transconductance (*g*_m_) equating to slope, and threshold *V*_*T*_, which corresponds to the *V*_*G*_-intercept. The linear fits were performed on the forward sweep of all the transfer curves. P50/50-6 with TFSI^–^ and BF_4_^–^ devices exhibited similar *g*_m_, whereas P50/50-6 with PF_6_^–^ were smaller by over an order of magnitude (Figure 7). The *V*_T_ of TFSI^–^ and PF_6_^–^ ranged between 1 and 2 V, whereas BF_4_^–^ devices consistently turned on below 1 V. These *V*_T_ values are significantly lower compared to BGBC devices made using the same poly(NDI2OD-T2) on SiO_2_ (*V*_T_ = 33.9 V, tested in air) which possessed similar charge trap densities (Table S4).^63^ This enabled the devices to operate at much lower operating voltages exhibited by the output curves (Figure S6).

The field-effect mobility (μ) was calculated from the *g*_m_ using the equation
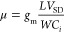
where *C*_*i*_ is the capacitance of the gate insulator and *L* and *W* are the length and width of the OTFT, respectively. The *C*_*i*_ value utilized corresponds to the EDL capacitance determined in the capacitor experiments at the same frequency. The resulting μ for P50/50-6 with TFSI^–^, PF_6_^–^, and BF_4_^–^ devices were 0.9 × 10^–3^, 1.1 × 10^–3^, and 1.1 × 10^–2^ cm^2^/(V·s), respectively. These values are similar to what we obtained using poly(NDI2OD-T2)-based BGBC for devices on SiO_2_ in air (3 × 10^–2^ cm^2^/(V·s)).^63^ The μs seen for devices from P50/50-6 with TFSI^–^ are comparable to our previous reports using poly(MMA-*r*-S)-*b*-poly(VBBI^+^TFSI^–^) with the same semiconductor.^15^ It has often been noted that a higher capacitance dielectric results in a reduced μ,^64,65^ which concurs with the greater μ obtained using devices fabricated using P50/50-6 with BF_4_^–^ compared to TFSI^–^. The reduced μ is mainly explained by referencing a microscopic model in which the charge carriers electrostatically accumulated at semiconductor surfaces undergo polaronic self-localization stemming from the interaction with polarizable dielectrics leading to Fröhlich polarons.^64,65^ This induces a broadening of the density of states (DOS) at the semiconductor/gating material interface and decrease in DOS at the Fermi energy, reducing the hopping probability and thus reduce μ.^66^ Previous reports by Fujimoto et al. using octathio[8]circulene (p-type semiconductor) based OTFTs with 1-butyl-3-methylimidozolium bis(trifluoromethylsulfonyl) (BMIM) cation based ionic liquids gating materials with different anions (TFSI^–^, BF_4_^–^, PF_6_^–^, TFS^–^) found a negative linear correlation between the μ and ionic liquid capacitance.^47^ For our devices, the ON/OFF ratios were relatively low (Table S4). These ON/OFF ratios could potentially be improved by reducing off currents which could be achieved by eliminating parasitic leakage currents outside the gate controlled area.^67^ Conventionally this is achieved by patterning the semiconductor layer, however, there have been relatively few reports involving the effective patterning of solution processed OTFTs.^68^ The authors also found that *V*_T_ significantly depends on the structure of ionic liquid gating medium, suggesting choice of ionic liquid is correlated to device performance. To the best of our knowledge, no similar study has been reported for n-type operation using ionic liquids or PILs but our results suggest a similar dependency between choice of anion and device performance.

Thus far, from a device performance perspective, P50/50-6 with BF_4_^–^ is the most suitable choice due to the resulting devices being characterized with relatively low *V*_T__,_ high *I*_SD_, and high μ (Figure 7). However, these experiments were run slowly to ensure a comparison of the devices once the EDL is formed and therefore do not properly characterize a device during intended operation. It is critical to study the device responsiveness to properly assess the potential of these materials. The responsiveness of the P50/50-6 (with either TFSI^–^, PF_6_^–^, or BF_4_^–^) gated OTFTs was examined by applying a 3 V square-pulse waveform to the gate at frequencies of 20, 100, 200, 600, 800, and 1000 mHz while measuring the *I*_SD_ (Figure 8). It is clear that the *I*_SD_ of the devices follow the input signal of the gate; however, it is also clear that the response changes with frequency and with choice of anion. For example: the use of P50/50-6 with TFSI^–^, the most conductive of the three, leads to devices that reach their maximum *I*_SD_ at very slow operations of 20 mHz and at fast operation of up to 1000 mHz, when we can see that the maximum is just barely reached within 0.5 s before the device is turned off. For devices fabricated using BF_4_^–^, the maximum *I*_SD_ is almost 5× greater than those made using TFSI- at low frequencies (<1000 mHz) but the *I*_SD_ reduces as the frequency is increased to a point where no current is detected at frequencies >800 mHz (Figure 8). We surmise this drop is due to the device not reaching saturation, that is, not being completely “on” before being switched “off”. For devices made using PF_6_^–^, the least conductive gating material, we only detected a current at slow operations <100 mHz. The difference in responsiveness is attributed to the ionic conductivity of the gating media; the device is turned on by the formation of an EDL, which form at lower frequencies for more conductive materials (as seen in the MIM section). The responsiveness, thus, follows the same order of increasing ionic conductivity with TFSI^–^ being the highest, followed by BF_4_^–^, then PF_6_^–^. What is interesting to note is that the TFSI^–^ containing materials led to more consistent current generation at all operating frequencies while the same polymer containing BF_4_^–^, (and possibly PF_6_^–^) led to a frequency-dependent *I*_SD__._

## Conclusion

In this study, we prepared a series of poly(styrene)*-b-*poly(1-(4-vinylbenzyl)-3-butylimidazolium-*random*-poly(ethylene glycol) methyl ether methacrylate) (poly(S)-*b*-poly(VBBI^+^[X]-*r*-PEGMA)) block copolymers, with varying PEGMA/VBBI^+^ ratios and three different mobile anions (where X = TFSI^–^, PF_6_^–^ or BF_4_^–^) to study structure–property relationships to gain insights toward designing materials for low-operating voltage and responsive OTFTs. We showed a library of PIL block copolymer materials, with two distinct *T*_g_ values exhibit self-assembled microstructures with long-range order and that the domain size and organizational structure depended on block copolymer architecture and choice of anion. We demonstrated that the use of these PIL-containing block copolymers leads to n-type OTFTs with comparable mobilities and very low threshold voltages (*V*_T_ as low as 0.5 V). By comparing identical polymers with different anions, we were able to probe the semiconductor/PIL interface during OTFT operation. We found that reduced glass transition temperatures and greater conductivity in the PIL block copolymers led to faster OTFT switching speeds. We have also shown that double-layer capacitance measured by EIS does not directly translate to device output currents even when the semiconductor/PIL interface is identical, suggesting OTFT fabrication is needed to screen potential PIL gating material candidates.

## Experimental Section

### Materials

BlocBuilder-MA was kindly donated by Marc Dubé (University of Ottawa), who received it from Arkema. *N*-*tert*-butyl-*N*-1-diethylphosphono-2,2-dimethylpropyl nitroxide (SG1) was synthesized following a procedure from Hlalele et al.^69^ 1,4-dioxane (99%), chloromethylstyrene (CMS, 99%), poly(ethylene glycol) methyl ether methacrylate (PEGMA, *M*_n_ = 300, 90%), styrene (99%), and dimethylsulfoxide (DMSO, 99%) were purchased from Sigma-Aldrich. 1-Butylimidazole (90%), lithium bis(trifluoromethanesulfonyl)amide (LiTFSI) (99%), lithium hexafluorophosphate (99%), and sodium tetrafluoroborate (98%) were purchased from Oakwood Chemical. Chromium (99.99%) and gold (99.99%) for electrodes were purchased from Angstrom Engineering (Kitchener, ON, Canada). Poly{[*N*,*N*-bis(2-octyldodecyl)-naphthalene-1,4,5,8-bis(dicarboximide)-2,6-diyl]-alt-5,5-(2,2-bithiophene)} (poly(NDI2OD-T2)), n-type semiconductor, was purchased from 1-Material (Montreal, QC, Canada). All solvents and regents were used as received unless specified otherwise.

### Synthesis of Poly(Styrene) Macroinitiator (Poly(S))

The poly(S) macroinitiator was synthesized by the polymerization of styrene (400 g, 3.84 mol) using BlocBuilder-MA (1.53 g, 4.00 mmol) with additional SG1 nitroxide mediating stable-free radical (117.7 mg, 0.4 mmol). The ratio of BlocBuilder-MA to styrene were selected to target a 100 kDa polymer at 100% conversion. The polymerization was performed in typical air-free conditions using a round-bottom flask fitted under a head of nitrogen with condenser. The reagents were first combined in the flask, the solvent was added (200 mL toluene), and the vessel was bubbled with nitrogen for 1 h. After it was purged, the vessel was heated to 100 °C. GPC samples were periodically taken to measure the *M*_w_ with an effort to stop the reaction at a rough molecular weight of 25 kDa corresponding to 25% conversion, to produce an effective, highly living macroinitiator. The reaction was stopped at 6 h, left to cool, then precipitated twice into methanol from tetrahydrofuran (THF). The final polymer was dried in vacuum, producing 112.5 g of the final poly(S) macroinitiator (*M*_n_ = 22.5 kDa, *M*_w_/*M*_n_ = 1.11).

### Synthesis of Poly(Styrene)*-b-*poly(chloromethyl Styrene*-r-*poly(ethylene glycol) Methacrylate) (Poly(S)-*b*-poly(CMS*-r-*PEGMA)) Precursor Block Copolymers

Precursor poly(styrene)*-b-*poly(chloromethyl styrene*-r-*poly(ethylene glycol) methacrylate) block copolymers were produced by performing a chain extension on the poly(S) macroinitiator. All of the formulations are listed in Table S1, and a typical procedure is as follows using the formulation CMS/PEGMA-50/50-4: poly(S) macroinitiator (3.00 g), CMS (1.35 g, 8.84 mmol), PEGMA (2.65 g, 8.84 mmol), and 50 wt % DMSO were loaded into a round-bottom flask sealed under a nitrogen condenser and purged with nitrogen for 20 min before heating to 90 °C. The reactions were left to run overnight. Final material was isolated in a similar fashion to the poly(S): twice precipitating to methanol, redissolving into THF between steps, and finally drying in vacuum overnight. Precursor polymers were characterized by NMR and SEC (Figures S1 and S2).

### Preparation of Poly(Ionic Liquid) Materials from Precursor Block Copolymers

Quaternization reaction were performed with a 1.1 molar excess of butylimidazole on the block copolymers containing CMS group (calculated by assuming entire polymer was CMS homopolymer) at 70 °C for 24 h, forming a poly(S)-*b*-poly(VBBI^+^Cl^–^-*r*-PEGMA)) block copolymers possessing a Cl^–^ mobile anion. The postreaction mixtures were then divided into three scintillation vials, and a subsequent anion exchange reaction was performed with a 1.1 molar excess of LiTFSI, LiPF_6_, or NaBF_4_ at room temperature for 48 h. The final poly(S)-*b*-poly(VBBI^+^[X]-*r*-PEGMA)) block copolymers (where X = TFSI^–^, PF_6_^–^, or BF_4_^–^) were all isolated by precipitating into water and thoroughly washed with water to remove any salts and finally dried in a vacuum oven, at 70 °C, overnight. The final poly(ionic liquid) block copolymers were characterized by ^1^H NMR to confirm quaternization was taken to full conversion and that the subsequent anion exchange was confirmed by shifting of neighboring protons on the imidazole group.

### Fabrication of Metal–Electrolyte–Metal (MEM) Capacitors

Metal–insulator–metal (MIM) capacitors were fabricated using one 25 mm × 25 mm glass slide as a substrate. The slides were first cleaned by sonication in soapy water, water, acetone and methanol for 5 min each, then blown dry with nitrogen. The cleaned slides were then patterned by physical vapor deposition (PVD) with bottom electrodes consisting of an initial 100 nm chromium adhesion layer, followed by 400 nm of gold. The final poly(S)-*b*-poly(VBBI^+^[X]-*r*-PEGMA)) block copolymers (where X = TFSI^–^, PF_6_^–^, or BF_4_^–^) were deposited by spin-coating 90 μL of solution (two thicknesses were targeted using either 60 and 100 mg/mL solutions) in methyl ethyl ketone (MEK) at 2000 rpm, followed by annealing at 130 °C for 2 h. Finally, top electrodes were deposited by PVD using a shadow mask; the pattern of the top and bottom electrodes results in 16 different capacitors on each slide with areas ranging from 0.35 to 2.88 mm^2^. For each poly(S)-*b*-poly(VBBI^+^[X]-*r*-PEGMA)) block copolymers (where X = TFSI^–^, PF_6_^–^, or BF_4_^–^), two substrates were prepared with different insulating layer thickness’ by changing the concentration of poly(ionic liquid) in MEK solutions. Capacitors were characterized by profilometry for thickness measurements and electrical impedance spectroscopy.

### Fabrication of Top-Gate Top-Contact (TGTC) Poly(Ionic Liquid)-Gated Organic Thin-Film Transistors

Top-gate top-contact organic thin-film transistors (OTFTs) were fabricated on top of 15 × 20 mm quartz coated glass substrates. First the semiconducting layer, poly{[*N*,*N*-bis(2-octyldodecyl)-naphthalene-1,4,5,8-bis(dicarboximide)-2,6-diyl]-alt-5,5-(2,2-bithiophene)} (poly(NDI2OD-T2)) was deposited by spin coating from a 10 mg/mL *o*-dichlorobenzene solution at 2000 rpm followed by annealing at 100 °C for 1 h. Next, 50 nm thick gold contacts were deposited for the source and drain electrodes using PVD aided by a shadow mask, with L = 30 μm and W = 1000 μm. Next, the poly(S)-*b*-poly(VBBI^+^[X]-*r*-PEGMA)) block copolymers (where X = TFSI^–^, PF_6_^–^, or BF_4_^–^) gating medium was deposited from spin coating from a 60 mg/mL solution in MEK at 2000 rpm The OTFTs were then annealed at 130 °C for 1 h to remove residual solvent and promote self-assembly of the block copolymer. A second layer of the poly(S)-*b*-poly(VBBI^+^[X]-*r*-PEGMA)) block copolymer was deposited by spin coating and annealed using identical conditions. The substrates were left to cool and finally completed by depositing a 50 nm thick gold gate electrode using PVD and shadow mask.

### Characterization

#### Size Exclusion Chromatography (SEC)

The molecular weight and dispersity of the poly(S)-*b*-poly(CMS*-r-*PEGMA) precursor polymers was determined by size exclusion chromatography. SEC was performed using an Agilent 1260 Infinity with columns heated to 30 °C with THF as the eluent at a flow rate of 1 mL·min^–1^. Wyatt detectors were utilized for the measurements; DAWN HELEOS II multiangle light scattering (MALS) detector, ViscoStar II differential viscometer, and an Optilab T-rEX differential index detector. The system was fitted with two MZ-Gel SD plus Linear 5 μm, 300 × 8.0 mm columns. The *∂n*/*∂c* for each material was determined using offline batch measurements with the RI detector, injecting a series of polymer in THF solutions of increasing concentration by a syringe pump and recording the dRI response. The dRI versus concentration was plotted, and the line of best-fit slope, equating to the d*n*/d*c*, was determined using a linear fit.

#### Nuclear Magnetic Resonance Spectroscopy (NMR)

The copolymer compositions and confirmation that polymer functionalization reactions were taken to completion was determined using nuclear magnetic resonance (^1^H NMR) using a Bruker AVANCE II 400 MHz spectrometer. Samples were dissolved in deuterated chloroform (CDCl_3_) or deuterated dimethyl sulfoxide, DMSO-*d*_6_, as the solvent. Determination of copolymer composition via ^1^H NMR is described in the Supporting Information.

#### Differential Scanning Calorimetry (DSC)

The glass transition temperatures (*T*_g_ values) of the final poly(S)-*b*-poly(VBBI^+^[X]-*r*-PEGMA)) block copolymer were determined using differential scanning calorimetry. DSC was performed using a TA Instruments Q2000 over a temperature range of −40 to 150 °C at a heating/cooling rate of 10 °C/min under an N_2_ environment. The *T*_g_ values were determined using the midpoint method from the second thermogram heating cycle using the software, Universal Analysis, provided by TA Instruments.

#### Profilometry

The thickness of the poly(S)-*b*-poly(VBBI^+^[X]-*r*-PEGMA)) block copolymer layer in MIM capacitors was measured using a Bruker Dektak XT profilometer. A series of 10 scratches were made with a diamond tip pen to create a step edge. The step edge was measured, and the average of the measurements ± SD are reported.

#### Electrochemical Impedance Spectroscopy (EIS)

Impedance properties, such as conductivity and double-layer capacitance, of the poly(S)-*b*-poly(VBBI^+^[X]-*r*-PEGMA)) block copolymers in the MIM capacitors were investigated with EIS. Measurements were conducted over a frequency range of 10^–3^–10^6^ Hz with an AC amplitude of 10 mV under atmospheric conditions using a Metrohm PGSTAT204. The ionic conductivity (σ) was calculated using the equation: σ = *d*/(*R*_b_*A*), where *d* is the thickness, *R*_b_ is the bulk resistance, and *A* is the area of the capacitor. Bulk resistance, *R*_b_, was found by using the diameter of a circular fit to the charge transfer regime, which appears as a semicircle, of the experimental data Nyquist plot of the experimental impedance data using Nova 2.1 software. A representative Nyquist plot with semicircular diameter fitting is shown in Figure S4.

#### Transmission Electron Microscopy (TEM)

Images of annealed bulk poly(S)-*b*-poly(VBBI^+^[X]-*r*-PEGMA)) block copolymer sample section were obtained using transmission electron microscopy. Samples were prepared by drop-casting 100 mg/mL polymer/MEK solution onto a Teflon watch-glass and left in a vacuum oven at 70 °C until solvent evaporated. The dried, cast polymer samples were then annealed at 130 °C under vacuum for 2 h and left to cool overnight. Liquid N_2_ was then poured over the annealed-polymer coated Teflon watch-glasses; the cast polymer was cracked, lifted from the watch-glass, and collected. Sections were cut on a Leica UCT ultramicrotome (Leica Microsystems, Leica UCT Ultramicrotome) equipped with a diamond blade, placed onto copper grids, and exposed to 5 wt % aqueous RuO_4_ solution for 1 h to preferentially stain the poly(styrene) block. TEM imaging was performed on the prepared samples using a FEI Titan 80-300.

#### Small-Angle X-ray Scattering (SAXS)

The SAXS patterns were collected with a Bruker AXS Nanostar system equipped with a Microfocus Copper Anode at 45 kV/0.65 mA, MONTAL OPTICS and a VANTEC 2000 2D detector. The distance, 106.95 cm from the detector to the sample, was calibrated with a Silver Behenate standard prior the measurements. The positioning fine-tuning was done by Nanography; a 2 s per step scan sweep on *X* and *Y* to find the exact position of the samples. The diffracted intensities were integrated from 0.100 to 3.000° 2θ on 360°. Collection exposures were 200–400 s

#### Transistor Characterization

Transistor characterization was performed in ambient conditions using a probe station equipped with a 2614B Keithley 2-channel source-measure unit (SMU) to control the gate and source–drain potentials, *V*_G_ and *V*_DS_, respectively. Output curves were generated measuring the source–drain current (*I*_SD_) and linearly sweeping *V*_DS_, while fixing *V*_GS_ at a fixed potential, performing a series of steps in *V*_GS_. Transfer curves were generated by fixing *V*_SD_ at 1 V and sweeping *V*_GS_. Frequency dependence of transfer characteristics were examined by measuring I_SD_ and using a function generator (AFG 3011C) to apply the gate voltage in a square-pulse waveform of selected frequencies. In the response time experiments, the gate potential was supplied by the waveform generator and measured using a Keithley Digital Multimeter (DMM 6500) to digitize the voltage. While the source–drain potential was set using one channel of the Keithley SMU connected in series with the device-under-testing and simultaneously measured the drain current, *I*_SD_.

## Supporting Information Available

Synthetic chemistry formulations and molecular weights for chloromethyl/poly(ethylene glycol) methyl ether methacrylate chain extension from poly(styrene) macroinitiator, along with representative characterization example; NMR spectra, determination of composition, and GPC trace; example Nyquist plot from polyelectrolyte capacitor with circular-best-fit used for conductivity calculation; detailed table of MIM capacitors including material, glass transitions, conductivity, and thickness; detailed transistor characterization results including; output curves, transfer characteristics (by the decade); and summary table with ON/OFF ratio, threshold voltage, capacitance, mobility, subthreshold swing, and charge trap density (PDF)
