# Supplementary material for: Ionic Liquid Containing Block Copolymer Dielectrics: Designing for High-Frequency Capacitance, Low-Voltage Operation, and Fast Switching Speeds
Source: JACS Au. 2021 Jun 9;1(7):1044–56. doi: 10.1021/jacsau.1c00133 (PMC8395628; doi:10.1021/jacsau.1c00133)
Supplement: Supplementary file 1 — au1c00133_si_001.pdf [file au1c00133_si_001.pdf]

Ionic Liquid Containing Block Copolymer Dielectrics: Designing for High Frequency Capacitance, Low-Voltage Operation and Fast Switching Speeds

Alexander J. Peltekoff[1], Samantha Brixi[1], Jukka Niskanen[1] and Benoît H. Lessard[1,2*]

[1]Department of Chemical & Biological Engineering, University of Ottawa, 161 Louis Pasteur, Ottawa, ON, Canada K1N 6N5

[2] School of Electrical Engineering and Computer Science, University of Ottawa, 800 King Edward, Ottawa, ON, Canada K1N 6N5

*Corresponding author: benoit.lessard@uOttawa.ca

SUPPORTING INFORMATION

Table S1. Formulations chloromethyl styrene/poly(ethylene glycol) methyl ether methacrylate chain extensions from poly(styrene) macroinitiator.

**Exp. ID** [a]	**CMS**		**PEGMA**	
	mmol	g	mmol	g
CMS/PEGMA-100/0-4g	26.21	4.00	-	-
CMS/PEGMA-100/0-8g	52.42	8.00	-	-
CMS/PEGMA-25/75-2g	1.90	0.29	5.70	1.71
CMS/PEGMA-25/75-4g	3.80	0.58	11.40	3.42
CMS/PEGMA-25/75-6g	5.70	0.87	17.10	5.13
CMS/PEGMA-50/50-4g	8.84	1.35	8.84	2.65
CMS/PEGMA-50/50-6g	13.26	2.02	13.26	3.98
CMS/PEGMA-75/25-4g	15.83	2.42	5.28	1.58
CMS/PEGMA-75/25-6g	23.75	3.62	7.92	2.38
CMS/PEGMA-75/25-8g	31.67	4.83	10.56	3.17

[a] Experimental identification (Exp. ID) is given by CMS/PEGMA-Y: where CMS and PEGMA correspond to chloromethyl styrene (CMS) and poly(ethylene glycol) methyl ether methacrylate (PEGMA), respectively. And the following number fraction refers to the molar feed of CMS to PEGMA, followed by the grams of total chain extension monomer.

[b] All experiments were performed utilizing the same amount of poly(styrene) macroinitiator (3.00 g).

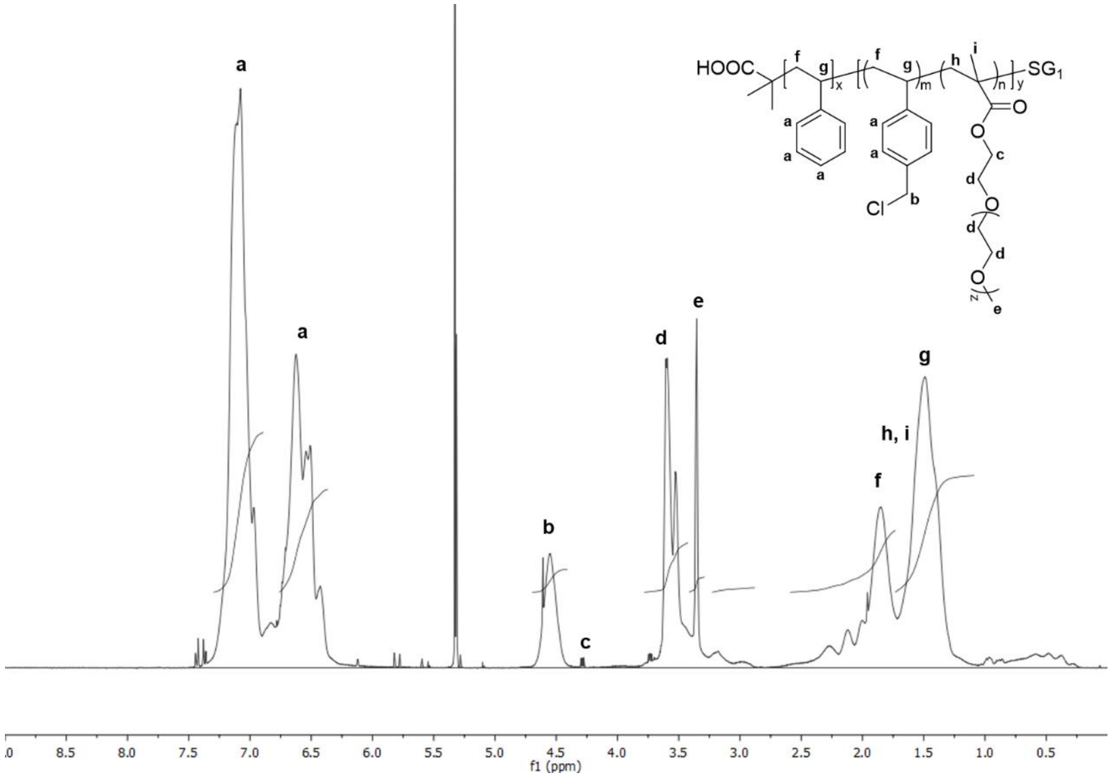

Figure S1. Representative ^{1}H NMR spectra of the precursor block copolymer in $CDCl_2$ (poly(S)-b-poly(CMS-r-PEGMA)-25/75-6g. Compositions are determined using the relative integrations of a, b, c, d, and e.

The molar ratio of Styrene, CMS, PEGMA was calculated:

$$ST : CMS : PEGMA$$

$$\frac{I_a - 2I_b}{5} : \frac{I_b}{2} : \frac{I_c + I_d + I_e}{21}$$

The composition of the entire polymer was calculated:

$$F_{ST} = \frac{ST}{ST+CMS+PEGMA}$$

$$F_{CMS} = \frac{CMS}{ST+CMS+PEGMA}$$

$$F_{PEGMA} = \frac{PEGMA}{ST+CMS+PEGMA}$$

The composition of the 2nd block was calculated:

$$F_{CMS} = \frac{CMS}{CMS+PEGMA}$$

$$F_{PEGMA} = \frac{PEGMA}{CMS+PEGMA}$$

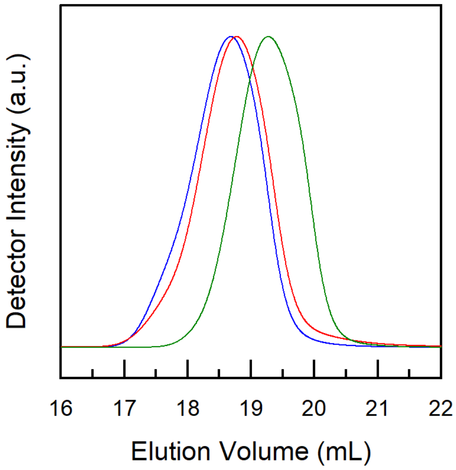

Figure S2. Representative SEC refractive index and light-scattering chromatograms of poly(styrene) macroinitiator (green) and poly(S)-b-poly(CMS-*r*-PEGMA)-100/0-8g (red) and poly(S)-*b*-poly(CMS-*r*-PEGMA)-25/75-4g (blue) diblock copolymers. The red line represents data from the light-scattering detector, and the blue line represents data from the RI detector.

Table S2. Molecular Weight Distribution Data for poly(S)-b-poly(CMS-r-PEGMA) Block Copolymer Precursors to Ionic Liquid Containing Block Copolymers Determined using poly(MMA) Standards.

Exp. ID	$\bar{M}_p$ (Da) $[kg\ mol^{-1}]$	$\bar{M}_w$ (Da) $[kg\ mol^{-1}]$
poly(S) macro.		
CMS/PEGMA-100/0-4g	37.3	39.5
CMS/PEGMA-100/0-8g	39.6	41.6
CMS/PEGMA-25/75-2g	41.8	43.4
CMS/PEGMA-25/75-4g	44.5	46.2
CMS/PEGMA-25/75-6g	40.2	40.9
CMS/PEGMA-50/50-4g	43.6	46.8
CMS/PEGMA-50/50-6g	39.2	43.7
CMS/PEGMA-75/25-4g	45.3	46.1
CMS/PEGMA-75/25-6g	42.8	45.9
CMS/PEGMA-75/25-8g	38.9	42.2

b) Number-average molecular weight ($\bar{M}_n$), weight-average molecular weight ($\bar{M}_w$), and dispersity (M_w/M_n) were determined by size exclusion chromatography (GPC). $\partial n/\partial c$ was determined by offline batch dRI measurements for each polymer

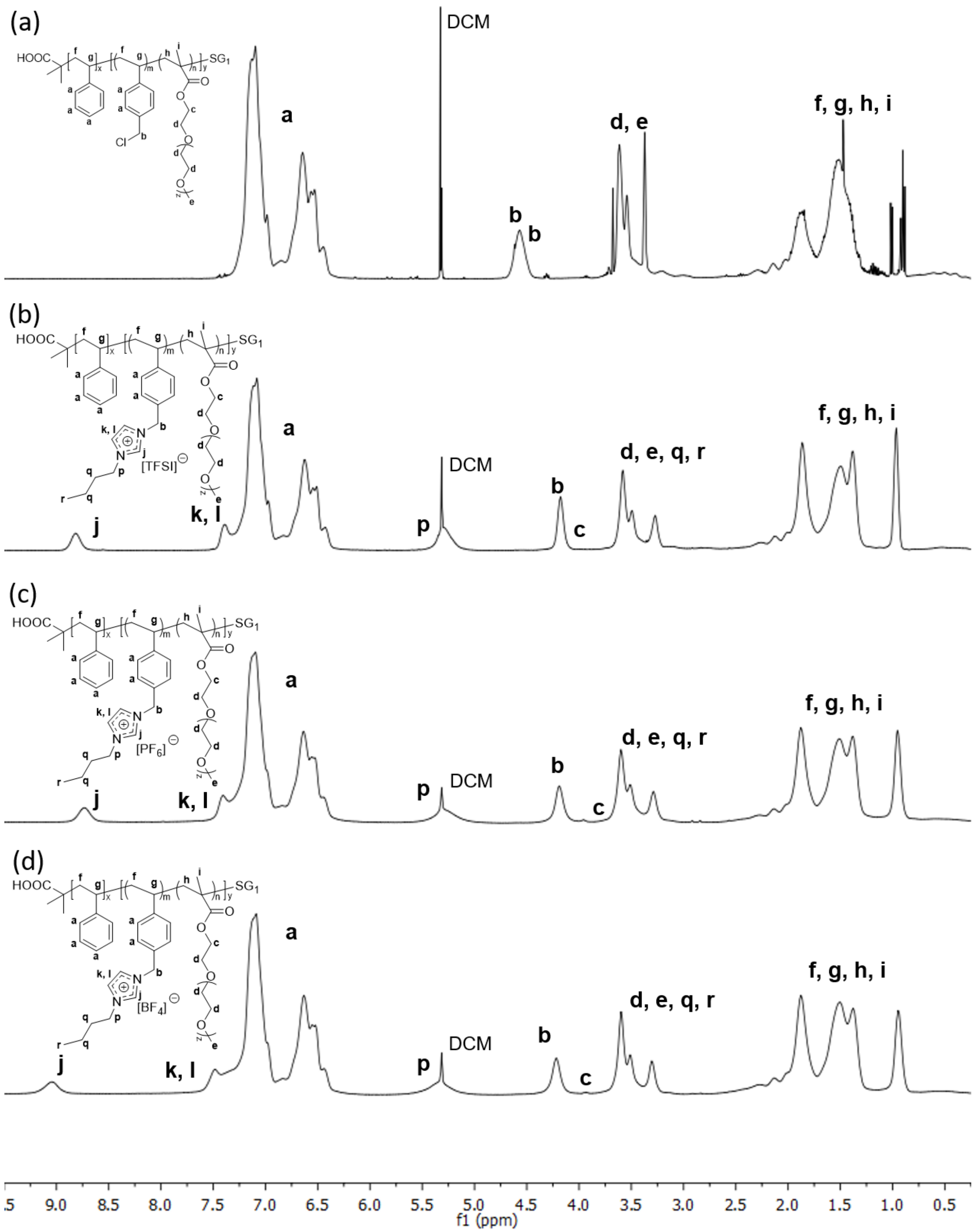

Figure S3. ^{1}H NMR spectra of (a) poly(S)-*b*-poly(CMS-*r*-PEGMA) precursor polymer, (b) poly(S)-*b*-poly(VBBI$^+$[X]-*r*-PEGMA)) block copolymers where X = TFSI$^-$, (c) poly(S)-*b*-poly(VBBI$^+$[X]-*r*-PEGMA)) block copolymers where X = PF_6^- anion, and (d) poly(S)-*b*-poly(VBBI$^+$[X]-*r*-PEGMA)) block copolymers where X = BF_4^- anion in $CDCl_2$. All polymers are from experiment CMS/PEGMA-75/25-8g.

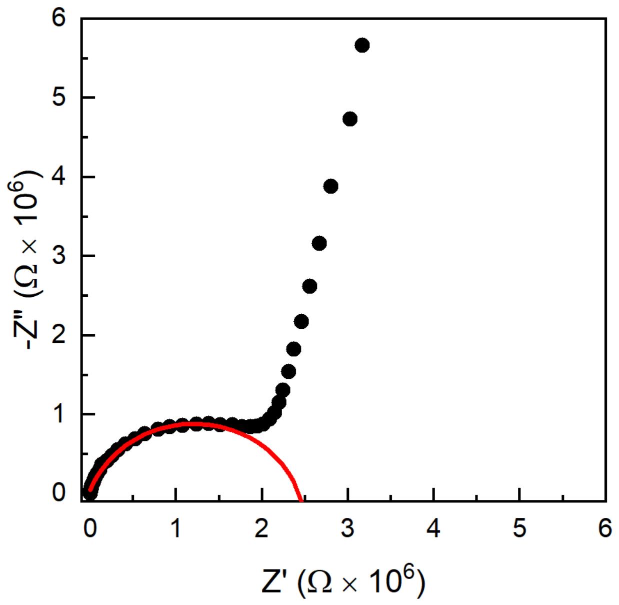

Figure S4. Representative Nyquist plot from metal-insulator-metal (MIM) capacitors. The conductivity was determined by performing a fit to the circular portion of the plot (red).

Table S3. Glass transition, conductivity, and profilometry data for poly(ionic liquid)s. Two capacitor sets of differing thickness were fabricated and characterized for each material.

EXP-ID	$T_{g,1}$	$T_{g,2}$	Conc. [mg/mL]	Thickness [nm]	Conductivity S cm^{-1}	Conc. 2 [mg/mL]	Thickness [nm]	Conductivity [S cm^{-1}]
poly(S) macro.	-	-	60	323 ± 24	-			
P100/0-4g-TFSI	105	12	60	427 ± 44	1.8×10^{-9}	100	1069 ± 107	3.3×10^{-9}
P100/0-4g-PF_6	101	-	60	481 ± 47	3.5×10^{-11}	100	935 ± 44	3.5×10^{-11}
P100/0-4g-BF_4	98	-	60	531 ± 244	-	40	285 ± 35	5.7×10^{-10}
P100/0-8g-TFSI	106	11	60	439 ± 22	1.3×10^{-7}	100	677 ± 68	1.4×10^{-7}
P100/0-8g- PF_6	104	-	60	480 ± 45	6.1×10^{-11}	100	794 ± 40	8.7×10^{-11}
P100/0-8g-BF_4	99	-	60	702 ± 47	7.7×10^{-9}	40	380 ± 13	1.5×10^{-9}
P25/75-2g-TFSI	99	-	60	444 ± 32	-	100	1069 ± 107	-
P25/75-2g-PF_6	99	-	60	416 ± 22	-	100	1138 ± 26	-
P25/75-2g-BF4	99	-	60	887 ± 59	-	100	1069 ± 107	-
P25/75-4g-TFSI	99	-	60	466 ± 32	2.5×10^{-8}	100	967 ± 59	4.6×10^{-8}
P25/75-4g-PF_6	99	-	60	486 ± 36	-	100	1085 ± 77	-
P25/75-4g-BF_4	99	-	60	560 ± 21	-	100	1513 ± 60	-
P25/75-6g-TFSI	99	-	60	399 ± 35	-	100	887 ±75	-
P25/75-6g-PF_6	99	-	60	381 ± 13	-	100	1081 ± 107	-
P25/75-6g-BF_4	99	-	60	401 ± 24	-	100	999 ± 85	-
P50/50-4g-TFSI	106	-7	60	421 ± 60	3.3×10^{-7}	100	756 ± 52	4.7×10^{-7}
P50/50-4g-PF_6	105	21	60	504 ± 57	1.8×10^{-9}	100	972 ± 62	3.0×10^{-9}
P50/50-4g-BF_4	105	17	60	989 ± 49	7.6×10^{-8}	40	262 ± 13	3.4×10^{-8}
P50/50-6g-TFSI	105	-7	60	430 ± 42	3.6×10^{-7}	100	682 ± 19	3.2×10^{-7}
P50/50-6g- PF_6	105	22	60	562 ± 80	4.9×10^{-9}	100	1031 ± 116	3.2×10^{-9}
P50/50-6g-BF_4	106	19	60	747 ± 95	1.4×10^{-7}	40	243 ± 17	1.7×10^{-8}
P75/25-6g-TFSI	107	2	60	382 ± 35	2.0×10^{-7}	100	849 ± 90	2.8×10^{-7}
P75/25-6g-PF_6	106	55	60	497 ± 69	5.9×10^{-11}	100	936 ± 284	5.8×10^{-11}
P75/25-6g-BF_4	105	48	60	847 ± 13	1.9×10^{-8}	40	231 ± 15	5.8×10^{-9}
P75/25-8g-TFSI	105	2	60	408 ± 119	1.4×10^{-7}	100	802 ± 75	1.9×10^{-7}
P75/25-8g-PF_6	105	52	60	591 ± 46	7.9×10^{-11}	100	919 ± 71	3.7×10^{-11}
P75/25-8g-BF_4	103	49	60	871 ± 54	2.0×10^{-8}	40	382 ± 17	4.2×10^{-9}

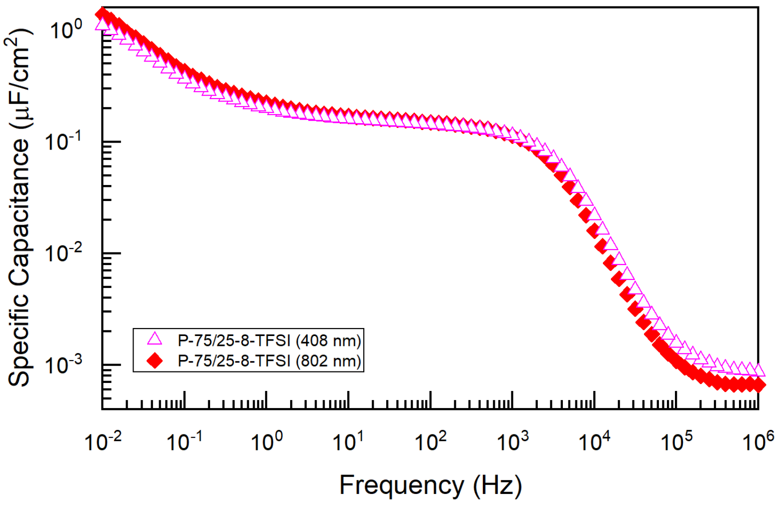

Figure S5. Characteristic Specific Capacitance versus Frequency curves for poly(S)-*b*-poly(VBBI$^+$[X]-*r*-PEGMA) (where [X] = TFSI$^-$) in MIM capacitors. Empty triangles represents MIM fabricated using 408 nm thick layer of PIL bloccopolymer, while the filled diamond represents MIM fabricated using 802 nm of the same PIL block copolymer

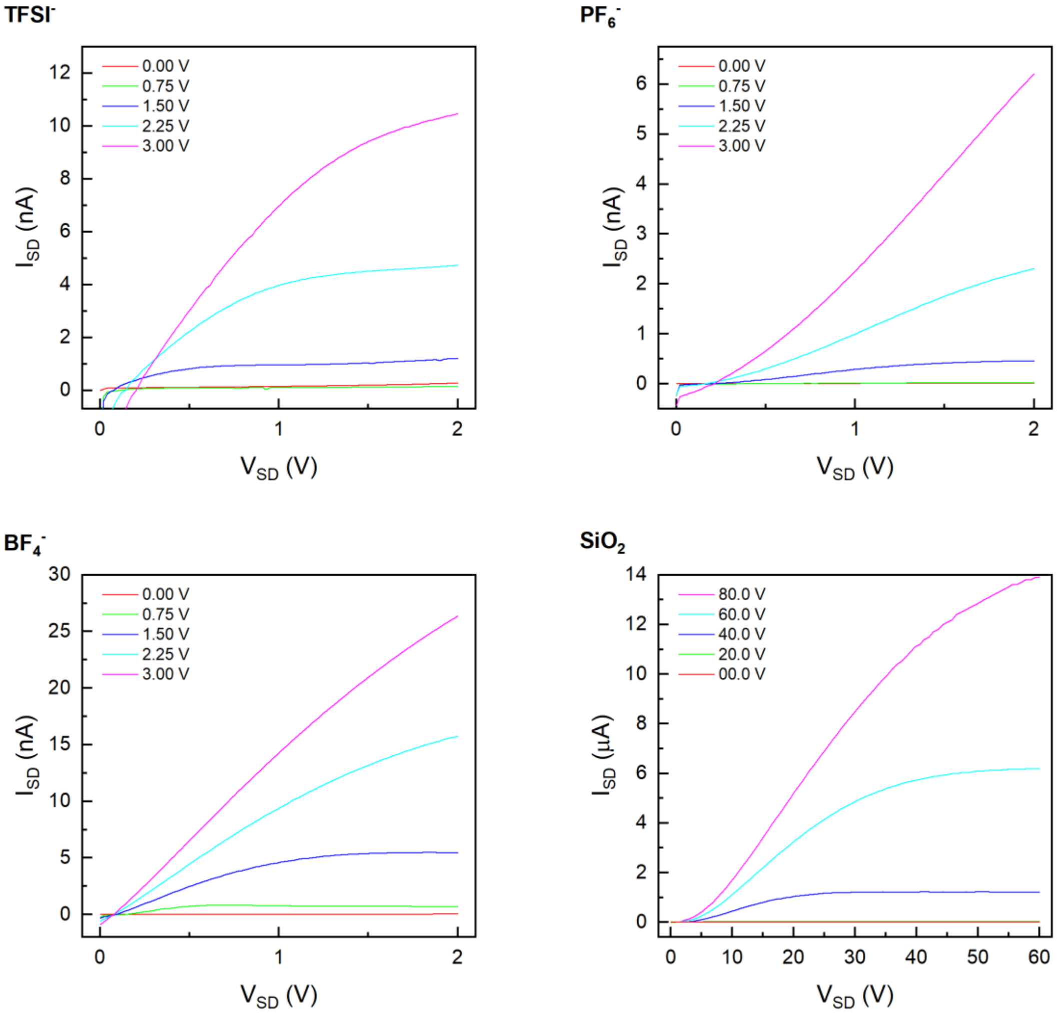

Figure S6. Representative output curves for TGTC devices with poly(NDI2OD-T2) n-type semiconductor using PF_6^-, BF_4^-, and $TFSI^-$ dielectrics. The dielectric layers ranged between 375 - 715 nm.

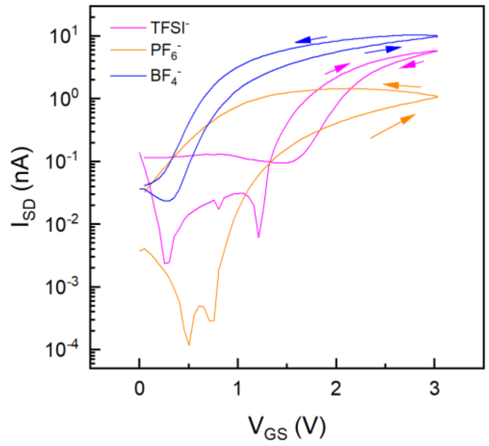

Figure S7. Representative transfer characteristics in the linear regime (V_{DS} = 1 V) of poly(S)-*b*-poly(VBBI$^+$[X]-*r*-PEGMA)) block copolymers (where X = $TFSI^-$, PF_6^- or BF_4^-) based OTFTs displayed with logarithmic scaling. The gate voltage was swept linearly at a rate of 250 mV/s. (L = 30 µm, W = 1000 µm)

Table S4. OTFT Characterization: ON/OFF, transconductance, threshold voltage, and field-effect mobility for each material.

Gating material[1]	ON/OFF[2]	Transconductance[3] [μSV^{-1}]	Threshold Voltage [V]	Capacitance[4] [Fcm^{-2}]	Mobility[5] [cm^{2}V^{-1}s^{-1}]	S_w [V/dec]	N_{it} [cm-2V-1]
$TFSI^-$	10^1	$(4.32 \pm 0.271) \times 10^{-8}$	1.5 ± 0.056	$(1.44 \pm 0.138) \times 10^{-6}$	$(9.0 \pm 1.03) \times 10^{-4}$	0.1	9.7×10^{12}
PF_6^-	10^1	$(5.87 \pm 0.557) \times 10^{-9}$	1.3 ± 0.093	$(1.60 \pm 0.687) \times 10^{-7}$	$(1.1 \pm 0.118) \times 10^{-3}$	0.5	8.3×10^{12}
BF_4^-	10^2	$(4.19 \pm 0.554) \times 10^{-8}$	0.7 ± 0.102	$(1.14 \pm 0.223) \times 10^{-7}$	$(1.1 \pm 0.234) \times 10^{-2}$	0.3	3.4×10^{12}
SiO_2[6]	10^2	-	33.9	1.50E-11	3.0E-2	5.1	8.0×10^{12}

1. All devices were fabricated from either SiO2 (taken from Brixi et al.[1]) or the P50/50-6 [X] material (where [X] = BF_4^-, PF_6^-, or $TFSI^-$)
2. ON/OFF ratios were obtained from the 100 mHz frequency plots
3. Transconductance obtained from the slope on the forward sweep of transfer curves
4. Obtained by EIS of capacitors fabricated with the poly(MMA-r-S)-b-poly(VBBI+[X]-r-PEGMA) (where [X] = BF_4^-, PF_6^-, or $TFSI^-$). Mean of the values summarized in Figure 6B.
5. Determined using device dimensions W/L = 1000µm/30µm
6. Calculated for a 230 nm SiO_2 dielectric thickness with dielectric constant = 3.9 using the equation: $C = \varepsilon\varepsilon_0 A/d$ where C is the capacitance, A is the area, ε is the dielectric constant, and ε_0 is the vacuum permittivity

References

[1] S. Brixi, O. A. Melville, B. Mirka, Y. He, A. D. Hendsbee, H. Meng, Y. Li, B. H. Lessard, *Sci. Rep.* **2020**, *10*, 1.
